# Impact of unhealthy food and beverage consumption on children’s risk of dental caries: a systematic review

**DOI:** 10.1093/nutrit/nuad147

**Published:** 2023-12-12

**Authors:** Jessica F Large, Claire Madigan, Rebecca Pradeilles, Oonagh Markey, Benjamin Boxer, Emily K Rousham

**Affiliations:** Centre for Lifestyle Medicine and Behaviour, School of Sport, Exercise and Health Sciences, National Centre for Sport and Exercise Medicine, Loughborough University, Loughborough, UK; Centre for Lifestyle Medicine and Behaviour, School of Sport, Exercise and Health Sciences, National Centre for Sport and Exercise Medicine, Loughborough University, Loughborough, UK; School of Sport, Exercise and Health Sciences, Loughborough University, Loughborough, UK; UMR MoISA (Montpellier Interdisciplinary Centre on Sustainable Agri-food systems), University of Montpellier, CIRAD, CIHEAM-IAMM, INRAE, Institut Agro, IRD, Montpellier, France; School of Sport, Exercise and Health Sciences, Loughborough University, Loughborough, UK; School of Sport, Exercise and Health Sciences, Loughborough University, Loughborough, UK; School of Sport, Exercise and Health Sciences, Loughborough University, Loughborough, UK

**Keywords:** children, dental caries, sugar-sweetened beverages (SSBs), systematic review, ultra-processed foods, unhealthy foods

## Abstract

**Context:**

The impact of unhealthy foods and beverages, namely those high in sugar, salt, and saturated or trans fats, has been studied extensively in relation to weight, body composition, and noncommunicable diseases, but less so in relation to the risk of dental caries. Few previous reviews have examined the evidence from all countries globally.

**Objective:**

A systematic review was conducted to assess the impact of unhealthy food and beverage consumption on the risk of dental caries in children aged ≤10 years, commissioned by the World Health Organization to inform updated complementary feeding recommendations.

**Data Sources:**

Systematic searches were conducted in the PubMed, Cochrane, and Embase databases for articles meeting the inclusion criteria dating from January 1971 to March 2022; supplementary searches were undertaken for articles from that period to June 2022.

**Data Extraction:**

Unhealthy foods and beverages were identified using nutrient- and food-based approaches. Risk of bias was assessed using the Risk Of Bias In Non-randomized Studies of Interventions (ROBINS-I).

**Data Analysis:**

A total of 30 023 unique citations were screened, yielding 37 studies for inclusion. Studies were conducted in high-income (n = 23 [62.2%]) or middle-income countries (n = 14 [37.8%]). Evidence synthesis was performed narratively, stratified by age (0 years to <2 years, 2 years to <5 years, and 5 years to ≤10 years) and exposure (unhealthy foods and unhealthy beverages). The heterogeneity of the exposures and comparators across studies was high. Almost all studies (n = 34) reported positive associations between the consumption of sugar-sweetened beverages or foods high in free sugars and dental caries. However, 67.6% of studies were assessed as having serious risk of bias.

**Conclusion:**

The evidence indicates that the consumption of unhealthy food and beverages in children ≤10 years appears to increase the risk of dental caries. Further longitudinal studies with high-quality dietary assessments, including studies in low-income countries and children aged >5 years at baseline, are recommended in order to build a more robust evidence base for use in the development of policy recommendations.

**Systematic Review Registration:**

PROSPERO registration no. CRD42020218109.

## INTRODUCTION

Overconsumption of unhealthy foods and beverages, namely those high in sugar, salt, and saturated or trans fats, is observed among children and is associated with poorer health outcomes.^1–3^ The global trend of increasing consumption of foods with high energy and low nutrient density, including refined foods such as sugary and savory snacks, is reflected across all socioeconomic groups.^4^ In the UK, around 9 in 10 children aged 1.5 years to 3 years exceed recommended daily sugar intake levels.^5^

Dental caries (dental decay) is the most common oral disease affecting children worldwide. It is estimated that 514 million children have experience of dental caries in primary (baby) teeth and 2 billion people have experience of dental caries in permanent (adult) teeth.^6^ Dental caries disproportionately affects children from more deprived communities, and this is compounded by a lack of access to oral health services.^6–10^

Free sugars include all monosaccharides and disaccharides added to foods by the manufacturer, cook, or consumer, plus sugars naturally present in honey, syrups, and unsweetened fruit juices.^11^ Frequent consumption of food and beverages containing free sugars is the main cause of dental caries among children and also increases the risk of obesity and other chronic health conditions, such as type 2 diabetes.^12–15^ Oral bacteria metabolize these dietary carbohydrates, ie, sugars, producing acids that demineralize dental enamel.^16–18^ Dental caries is associated with difficulty eating, disturbed sleep, school absence, and emotional distress among children. Untreated dental caries can have a negative impact on quality of life, and growth and development.^19–23^

A previous systematic review undertaken to inform the World Health Organization (WHO) guidelines on the association between free sugar intake and dental caries, and the effect of restricting sugar intake searched the published literature up to 2011.^18^ Fifty-five studies met the eligibility criteria, with the majority (24) being cross-sectional studies.^18^ In the studies of children, 42 out of 50 reported at least 1 positive association between sugar intake and dental caries.^18^ Moderate-quality evidence demonstrated a lower caries prevalence when free sugar intake was <10% of the total energy intake (EI), and a significantly lower prevalence of caries when free sugar intake was <5% of EI, although the quality of the evidence for the latter was reported as very low.^18^

In more recent studies, the impact on children’s oral and general health from the consumption of free sugars in the form of sugar-sweetened beverages (SSBs) has been explored.^15^^,^^24–26^ SSB consumption by children has tripled over the past 50 years, and this trend has been associated with negative health outcomes and an increasing prevalence of noncommunicable diseases, including dental caries, overweight/obesity, type II diabetes, and cardiovascular disease.^26^ Studies exploring the association between SSBs and weight are much greater in number than those on the impact of SSBs on oral health.^26^^,^^27^ In those studies that do investigate oral health and SSBs, the majority of the evidence is cross-sectional, providing weak evidence of associations because of the potential for reverse causality.^18^^,^^28^ This review excludes cross-sectional data in recognition of the need for further research involving longitudinal and interventional studies.^28^^,^^29^

Overall, there is a relative paucity of research on the association between dental caries and the consumption of unhealthy foods and beverages, especially in low- and middle-income countries, with prior research largely focusing on SSBs or free sugars.^18^^,^^30^^,^^31^ Hence, the aim of this systematic review was to examine, among children aged ≤10 years, the risks of greater consumption of unhealthy foods and beverages compared with low or no consumption, with specific reference to dental caries. This systematic review provides a comprehensive assessment of the impact of all unhealthy foods and beverages, including assessment of frequency of intake using a food-based approach and not solely the impact of nutrients such as total sugar intake on dental caries. This systematic review was part of a wider review commissioned by WHO to inform updated complementary feeding guidelines.

## METHODS

This systematic review was registered (CRD42020218109, accessible at https://www.crd.york.ac.uk/PROSPERO) and is reported according to the preferred reporting items for systematic reviews and meta-analyses (PRISMA) statement.^32^^,^^33^

### Eligibility criteria

Study eligibility was based on the inclusion and exclusion criteria for population/participants, interventions or exposures, comparators and outcomes (PI/ECO) shown in Table 1. The PI/ECO for this commissioned systematic review was specified by the WHO, including the age criteria for participants/populations (≤10 years at exposure or intervention). The exposure was high consumption of unhealthy foods and beverages vs low or no consumption (comparator). The outcome was dental caries. Reports published from January 1971 onwards were included. No restriction on language was applied. Non-English-language reports were screened by native speakers with subject-specific knowledge of nutrition or health-related studies.

**Table 1:** PICOS criteria for inclusion and exclusion of studies

Parameter	Inclusion criterion	Exclusion criterion
**Participants/population**	Children’s age at intervention or exposure: infants from birth to ≤10.9 years	Age at intervention or exposure >10.9 years Studies that exclusively enroll participants with a disease Studies using hospitalized patients, severely malnourished participants, or clinical populations Studies of exclusively pre-term babies (<37 weeks gestation) or exclusively babies that are low birth weight (<2500 g) or small-for-gestational age
**Independent variable (intervention or exposure)**	Studies reporting (greater) consumption of unhealthy foods and beverages compared with no or low consumption	Studies not reporting consumption of unhealthy foods and beverages as per the protocol definition of consumption
	Unhealthy foods defined using (i) nutrient-based approaches (foods high in added sugars, free sugars, artificial sweeteners, fats [eg, saturated/trans], salt); and food-based approaches, including (ii) ultraprocessed foods (based on NOVA classification, excluding formula and follow-on milks); (iii) unhealthy foods and beverages listed in the WHO and UNICEF guide (2021); (iv) food items defined by authors using terms such as “fast-food,” “convenience foods,” or “non-core foods”	Studies reporting only dietary patterns (ie, data reduction techniques such as Principal Component Analysis) or eating practices (eg, meals per day; snacking patterns; meal times, and duration of eating episodes)
	Consumption defined as: (i) quantities consumed (g/d, wk, or month); (ii) portion sizes; (iii) frequency of consumption (per wk, month, y), or consumed/non-consumed	
**Comparator**	Consumption of less or no unhealthy foods and beverages: no or low added sugar, free sugars, artificial sweeteners; less fat (or less of certain types of fat), less consumption of foods high in salt or ultraprocessed/energy-dense, nutrient-poor foods	
**Study design**	Randomized controlled trials (RCTs) Non-RCTs (including historically controlled studies) Prospective cohort studies (including interrupted time series analyses) Retrospective cohort studies Pre/post studies with a control	Cross-sectional studies Trials without a control group Narrative reviews, systematic reviews, and meta-analyses Case–control studies (ie, cases with disease [eg, diabetes] vs controls without disease) Pre/post studies without a control
**Dependent variable (outcome)**	Dental caries	
**Publication status**	Reports published in peer-reviewed journals	Conference abstracts, conference proceedings, unpublished data, reports, letters, editorials

Unhealthy foods and beverages were identified using food-based as well as nutrient-based approaches and included: foods defined as ultraprocessed using the NOVA classification^34^; unhealthy foods and beverages defined by WHO and the United Nations Children’s Fund (UNICEF) infant and young child feeding indicators, which include sweetened beverages (commercially or home-prepared drinks, including fruit juices, and sentinel unhealthy foods [ie, sweet foods and fried/salty foods].^35^ Other foods considered unhealthy were those high in added sugars, free sugars,^36^ artificial sweeteners, salt, or rich in saturated and/or trans fats. Finally, studies were included in which authors used terms denoting unhealthy foods such as “junk foods”, “fast foods”, or “convenience foods”. Comprehensive details of the classification of unhealthy foods and beverages are provided in Table 2.^35–37^

**Table 2:** Classification used to identify foods and beverages as unhealthy for the review of effects of unhealthy food and beverage consumption among children aged ≤10 years on risk of dental caries

**A. Unhealthy foods and beverages (ultraprocessed foods) defined as per the NOVA classification system** ^a^
Sugar-sweetened beverages (sweetened fruit and vegetable juices, soft drinks, fruit and vegetable concentrates, fruit-flavored drinks, fruit and vegetable smoothies, nectars, chocolate/cocoa drinks, milk/yoghurt drinks, energy drinks, sweetened/flavored water). These refer to packaged/commercially produced drinks.
Diet or light soft drinks (with non-caloric or artificial sweeteners)
Fruit/flavored/sweetened yoghurts
Chocolate
Candies/sweets
Ice cream
Sweet packaged snacks (eg, sweetened popcorn, caramelized nuts)
Savory packaged snacks (eg, crisps, salted popcorn, cheese puffs)
Margarine and other spreads
Biscuits
Pastries (eg, croissant, pain au chocolat, brioche, doughnuts)
Energy bars
Cakes
Sweetened breakfast cereals
Instant noodles
Pizza
Pies
Processed meat or reconstituted meat products (eg, sausages, ham, hot dogs, fried/battered chicken, poultry nuggets) and fish nuggets/battered fish
**B. Unhealthy foods and beverages items defined in the WHO-UNICEF sentinel unhealthy food categorization** ^b^ (including only those items not already listed under A)
Fried potatoes/chips
100% fruit juices (ie, unsweetened), whether made at home, by informal food vendors or packaged in cans, bottles, boxes, or sachets, and other sweet beverages that are home-made and to which any kind of sweeteners (eg, sugar, honey, syrup, or flavored powders) have been added
**C. Unhealthy items defined as high in saturated fat content** (including only those items not already listed under A or B)
Butter, lard, ghee
**D. Unhealthy items defined as high in free sugar content** ^c^ (including only those items not already listed under A, B, or C)
Table sugar
Jam, honey, syrups
Unsweetened, 100% fruit and vegetable juices, concentrates, and smoothies
**E. Other included terminologies used by study authors to refer to unhealthy items**
Non-core food; extra food; convenience foods; junk food; fast food; snack foods

a NOVA classification based on Monteiro et al. 2010.^37^

b Based on WHO and UNICEF, 2021.^35^

c Based on Swan et al. 2018.^36^

### Searches

A search for studies published from 1971 to December 23, 2020 in the Cochrane Library, Embase, and PubMed databases was undertaken. Updating database searches continued until March 10, 2022. Supplementary searches continued until June 30, 2022, including hand searches of the lists of references of included reports and relevant published systematic reviews, and consultation with subject experts regarding relevant published studies that had not been identified from database searches. The search strategies for each database are included in Table S1 in the Supporting Information online.

### Study selection

The results were uploaded to Covidence software (Veritas Health Innovation), and duplicates were removed. Two independent reviewers from the wider review team (E.K.R., R.P., P.G., S.G., O.M., M.S., K.B., N.P., J.L., and C.M.) screened each study title, abstract, and full text. Disagreements were discussed and resolved by a third reviewer (E.K.R., R.P., J.L., or C.M.). All decisions were recorded in Covidence, and the reviewers were blinded to each other’s decisions. One reviewer undertook the data extraction for each report, working independently (E.K.R., B.B., or J.L.). Any data extraction queries were discussed among the team. A second reviewer checked 50% of all records extracted for completeness and accuracy (E.K.R. or J.L.).

### Outcomes and summary measures

Data were extracted for the baseline and follow-up periods for all included studies. Where multiple follow-up assessments were reported, we extracted the data for all time points. Food consumption data were extracted as the quantity consumed (eg, g/d), frequency of consumption, or whether the item was consumed or not. Data were extracted from all the reports of a single study if they presented unique data, different outcomes, or different exposures. If 2 reports presented the same outcomes and exposures, data was extracted from the report that most directly addressed the systematic review question, to avoid duplication.

### Effect measures

Measures of intervention effect (odds ratios [ORs], adjusted odds ratios [aORs], beta coefficients, relative risks with 95% confidence intervals, or *P* values) were extracted from all studies providing data on the effect of the exposure on the outcome of interest. Data were extracted from fully adjusted models where available. If unadjusted effect measures only were reported, these were extracted. Unadjusted estimates were included without recalculations, due to the constraints on time and resources for the review.

### Synthesis

Findings were synthesized using the PICO framework of participants (P), intervention (I), comparator (C), and outcome (O), first grouping studies by study design, and intervention/exposure, then by participant characteristics. Exposures were stratified into unhealthy beverage consumption and unhealthy food consumption. Participant characteristics were stratified by the following age groups: 0 years to <2 years; 2 years to <5 years and 5 years to ≤10 years.

Measures of effect were tabulated based on the availability and type of data. For completeness, all estimates are included in summary tables of results, including studies with critical risk of bias. In the narrative synthesis, however, the results were not reported from studies assessed as having critical risk of bias, in line with the existing guidance.^38–40^ Exposures and outcomes were cross-tabulated for potential meta-analysis, but the level of heterogeneity across the studies was too great for a meta-analysis to be conducted. The heterogeneity arose from the variety of assessment tools used for dental caries, the different ways of reporting dental outcomes, variation across the dietary exposures, and different ways of reporting the frequency or quantity of consumption, which together prevented these data from being harmonized for meta-analysis.

### Risk-of-bias assessment

Risk of bias was assessed independently by 2 reviewers (2 of E.K.R., B.B., and J.L.), within the Covidence software, ensuring blinding of the independent reviewers. If agreement could not be reached, a third reviewer assessed the risk of bias and a consensus was reached. Risk of bias was conducted at the outcome level.

The Risk Of Bias In Non-randomized Studies of Interventions (ROBINS-I) tool was applied, following Cochrane guidance.^39^^,^^41^ Each of the 7 domains in the risk-of-bias tool was rated as having low, moderate, serious, or critical risk of bias, or no information.^39^ For bias due to confounding (Domain 1), in line with guidance, an observational study that has controlled for important confounding variables should be judged as having moderate risk of bias.^39^ This was applied to the consumption of unhealthy food and beverages (the intervention), which were considered nonrandom within a population, even after controlling for important confounders. A rating of moderate for this domain indicates the study is considered sound as a nonrandomized study but is not entirely comparable with a well-conducted randomized trial.^39^ A low risk of bias is applicable only to a study in which no confounding is expected.^39^ Studies that omitted 1 or more important confounding variables, or in which the reliability or validity of measurement of an important aspect was low enough to lead to serious residual confounding, were assessed as being at serious risk of bias.^39^ Studies that did not control for any confounding variables (eg, studies using univariable analyses) were assessed as being at critical risk of bias for this domain. Important confounding variables were baseline status for dental caries, age, sex, socioeconomic status, maternal/parental education, fluoride exposure, dental care, oral hygiene practices, and oral health knowledge and attitudes. After obtaining consensus on the seven domains of the tool, the overall risk of bias (low, moderate, serious, critical, or no information) for each study was assessed using the criteria in Table S2 in the Supporting Information online.^39^

## RESULTS

### Study selection

**Figure 1:**
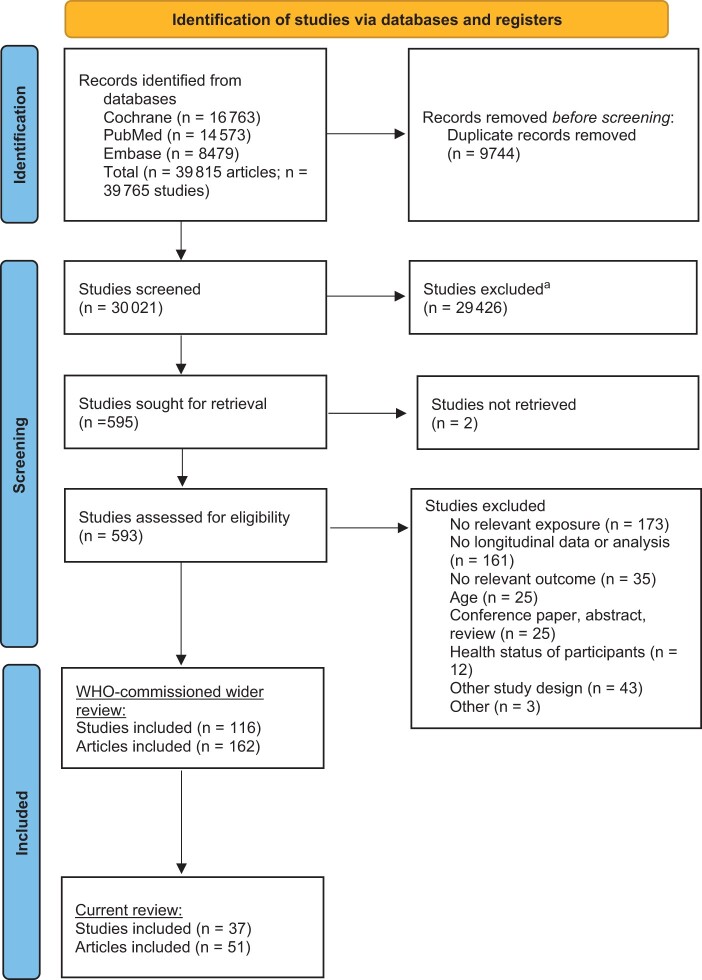
**Flow diagram of the literature search selection process**. ^a^No automation tools were used; all screening was undertaken by the review team. *Source*: Page et al (2021).^32^

The search for the wider systematic review retrieved 39 815 reports of which 9744 duplicate records were removed (Figure 1^32^). A total of 30 021 reports were screened, of which 595 were eligible for full-text review. Of these, 593 records were assessed for eligibility, as 2 of the reports could not be retrieved at full-text stage.^42^^,^^43^ Three articles in Chinese language were included.^44–46^ All other included reports were in English language. A total of 30 studies were eligible for inclusion. In the updated search, 3479 reports were screened, of which 38 were eligible for full-text review. Seven further studies were eligible for inclusion in the review. Overall, 37 studies (51 articles) reporting dental outcomes were included in this review.

### Study characteristics

The characteristics of the 37 included studies are summarized in Table 3.^44–94^ The year of publication of the studies extended from 1991 to 2022. Almost two thirds of studies (n = 23, 62.2%) were conducted in high-income countries, and over one third (n = 14, 37.8%) in middle-income countries, based on the current Gross National Income per capita.^95^ Studies in middle-income countries were conducted in China, Brazil, Thailand, South Africa, and Iran. No studies were conducted in countries currently listed as low-income. Most studies were prospective cohort studies, with no randomized controlled trials. Twenty-one studies (56.8%) stated that the participants were from urban settings; 5 studies (13.5%) recruited from both rural and urban areas, and only 1 study (2.7%) recruited from rural areas only. Ten studies (26.3%) did not specify the residence/location of participants. The sample size of the included studies ranged from 93 to 31 202.^63^^,^^92^

**Table 3:** Characteristics of included studies reporting on effect of unhealthy food and beverage consumption and dental caries outcomes in children aged ≤10 years

Study ID	Reference	Country	Setting (R/U)	Income level^a^	Recruited from	Exposure	Baseline age (mean or range)	Outcome assessed
Anderson 2021	Anderson et al 2021^47^	Sweden	U	HIC	Clinic	Sweets and sugar-containing beverages	1 y	ICDAS 1–6; defs; DFS
Bernabe 2020	Bernabe et al 2020^48^	Scotland	U	HIC	Clinic	SSB	12.8 mo	dmfs
Chaffee 2015	Chaffee et al 2015^49^	Brazil	U	UMIC	Clinic	6 mo sweet index; 12 mo sweet index	6 mo	Severe ECC; dmft
De Melo 2019	De Melo et al 2019^50^	Brazil	U	UMIC	Clinic	Sweets	30 mo	dmft index
Devenish 2020	Devenish et al 2020^51^	Australia	U	HIC	Clinic	Energy as free sugars	3 mo	Presence of ECC (dmfs)
Echeverria 2022	Echeverria et al 2022^52^	Brazil	U	UMIC	Pelotas Birth Cohort	Trajectory of sugar consumption from 3 mo to 48 mo	Not recorded	ICDAS and dmfs
Feldens 2010	Feldens et al 2010^53^	Brazil	U	UMIC	Clinic	High density of sugar	6 mo	Severe EEC (dmfs) at 4 y
	Feldens et al 2021^54^	Brazil	U	UMIC	Clinic	Sugar-containing items; household sugar purchase	6 mo (sugary food purchase), 3 y (household sugar purchase)	dmft
Grindefjord 1996	Grindefjord et al 1996^55^	Sweden	U	HIC	NS	Sugar-containing beverages; candy	30 mo	Initial/manifest dental caries (chalky appearance [smooth surface] and detection on probing [occlusal surface])
Hao 2015	Hao 2014^44^	China	U	UMIC	Clinic	Sweets/candies	3 y	dmfs
	Hao et al 2015^56^	China	U	UMIC		Sweets/candies	3 y	dmfs
Holt 1991	Holt 1991^57^	UK	U	HIC	NS	Sweetened snacks or drinks	2 y	dmft
Hooley 2012	Hooley et al 2012^58^	Australia	Both	HIC	Home	Sweet drinks; high-fat foods	4.79 y	“Dental problems” [cavities, fillings, extractions] reported by primary caregiver, 6 y–7 y and 8 y–9 y
Ismail 2008	Ismail et al 2008^59^	USA	U	HIC	Home	Soda beverages	0 y–5 y	ECC; Severe ECC (dmfs and ICDAS)
	Ismail et al 2009^60^	USA	U	HIC		Soda beverages	2.6 y	Caries increment
	Lim et al 2015^61^	USA	U	HIC		Soda beverages	0 y–5 y	dmfs
	Lim et al 2019^62^	USA	U	HIC		Soda beverages	0 y–5 y	Dental caries
Jordan 2020	Jordan et al 2020^63^	USA	R	HIC	Community centers	Juice	8 mo–18 mo	dmfs
Lin 2022	Lin et al 2022^64^	Taiwan	National	HIC	Schools	SSBs	8 y–9 y	Caries at cavitation level (WHO) and dmft, DFMT, and DFMS
Lopes-Gomes 2021	Lopes-Gomes et al 2021^65^	Brazil	U	UMIC	NS	Snacks containing sugar	1 y–3 y	ICDAS
MacKeown 2000	MacKeown et al 2000^66^	South Africa	U	UMIC	NS	Added sugar	1 y	dmfs incidence
Mahboobi 2021	Mahboobi et al 2021^67^	Iran	U	LMIC	School	Sugary snacks	7 y–8 y	CAST index
Manohar 2021	Manohar et al 2021^68^	Australia	U	HIC	Secondary analysis	Discretionary food^4^	4 mo	dmfs
Marshall 2003	Chankanka et al 2011^69^	USA	NS	HIC	Clinic	Meals and snacks: beverages, fruit juices, soda, sports drinks, desserts, candy, added sugar, processed starch foods	5.0 y	Caries detected by visual and tactile methods (probe)
	Chankanka et al 2015^70^	USA	NS	HIC		Sodas, juice drinks, and 100% fruit juice	5.1 y	dmfs
	Curtis et al 2018^71^	USA	NS	HIC		SSB and 100% juice	7 y–9 y	dmfs
	Levy et al 2003^72^	USA	NS	HIC		Pop/sports drink consumption at 12 mo–24 mo; pop/sports drink consumption at 36 mo–48 mo; sugar beverages at 12 mo–24 mo	6 wk	d_1_ lesions; d_2–3_ lesions
	Marshall et al 2003^73^	USA	NS	HIC		Regular soda pop; sugar-containing powdered beverages	1 y	d_1_ lesions; d_2–3_ lesions
	Warren et al 2002^74^	USA	NS	HIC		Soft drinks; juice	4.7 y	Tooth wear
Mattila 2005	Mattila et al 2001^75^	Finland	Both	HIC	Clinic	Sweets	3 y	Dental caries: dmfs score
	Mattila et al 2005^76^	Finland	Both	HIC		Sweets; daily sugar consumption	18 mo	dmft/DMFT score at 10 y
Mei 2021	Mei et al 2021^77^	China	U	HIC	Nurseries	Carbonated beverages; sweet snacks	3 y–4 y	dmft, dmfs
Meurman 2010	Meurman and Pienihäkkinen 2010^78^	Finland	U	HIC	Clinic	Added sugar; sweet snacks	18 mo	dmft; ECC
Park 2015	Park et al 2015^79^	USA	NS	HIC	Mail	SSB	10 mo–12 mo	Number of dental cavities child by age 6 y had experienced, reported by caregiver
Pang 2015	Pang et al 2015^45^	China	Both	UMIC	School	Soda drinks; cookies and sweet breads	3 y–6 y	DMFT/dmft caries
Peltzer 2014	Peltzer et al 2014^80^	Thailand	NS	UMIC	Clinic	Sweet candy	24 mo	dmft and dmfs
	Peltzer and Mongkolchati 2015^81^	Thailand	NS	UMIC	Clinic	Sweet food index	30 mo	Severe ECC
Peres 2016	Peres et al 2016^82^	Brazil	U	UMIC	Clinic	Sugar intake	1 mo	dmft score
Rodrigues 2000	Rodrigues and Sheiham 2000^83^	Brazil	U	UMIC	School	Sugary food	3 y	Change in dmfs
Karjalainen 2015	Karjalainen et al 2001^84^	Finland	U	HIC	Clinic	Sweet intake	37.4 mo	dmft
	Karjalainen et al 2015^85^	Finland	U	HIC		Added sucrose (sucrose and other free sugars)	3 y	dmft/DMFT
	Ruottinen et al 2004^86^	Finland	U	HIC		Sucrose-containing foods	13 mo	dmft and DMFT
Sakuma 2007	Sakuma et al 2007^87^	Japan	NS	HIC	Clinic	SSB; sweets	1.5 y	Change in caries
Skafida 2018	Skafida and Chambers 2018^88^	UK	NS	HIC	Child Benefits Register (random sample)	Soft drinks; sweets/chocolate	2 y	Dental decay (decayed, extracted, or filled teeth)
Tamaki 2009	Tamaki et al 2009^89^	Japan	U	HIC	School	Sweet juice; sweet snacks	5 y or 6 y	Incident caries (baseline to follow-up): dmft, and classification by Ministry of Health and Welfare, Japan
Thornley 2021	Thornley et al 2021^90^	New Zealand	Both	HIC	Clinic	Sugary soft drinks; fruit juice; confectionary/cakes; noodles/rice porridge; ice-cream; takeaways	2 y	dmft
Warren 2009	Warren et al 2009^91^	USA	NS	HIC	Clinic	SSB	6 mo–24 mo	Cavitated and non-cavitated dental lesions (classification not named)
Watanabe 2014	Watanabe et al 2014^92^	Japan	NS	HIC	Clinic	SSB; sweet snacks	1.5 y	Dental caries (visual and probe)
Wigen 2015	Wigen and Wang 2015^93^	Norway	NS	HIC	Mail	SSB	1.5 y	sum of dmft
Winter 2015	Winter et al 2015^94^	Germany	NS	HIC	School	Sugar index	3.5 y	dmft increment
Wu 2020	Wu et al 2020^46^	China	Both	MIC	School	Candy	4.2 y	dmft rate

a Calculated using the World Bank Atlas method for the 2021 fiscal year (based on gross national income per capita in 2019), https://datahelpdesk.worldbank.org/knowledgebase/articles/906519-world-bank-country-and-lending-groups. *Abbreviations*: defs, decayed with manifest caries, extracted, and filled surfaces; dmfs, decayed-missing-filled surfaces (for primary teeth); DFS, decayed-filled surfaces (for permanent teeth); DMFS, decayed-missing-filled surfaces (for permanent teeth); dmft, decayed-missing-filled teeth (for primary teeth); DMFT, decay-missing-filled teeth (for permanent teeth); d1, non-cavitated lesions; d2–3, cavitated lesions; ECC, early childhood caries; HIC, high-income country; Hb, hemoglobin; ICDAS, International Caries Detection and Assessment System, a clinical scoring system, range 1–6; MIC, middle-income country; NS, not stated; R, rural; RCS, retrospective cohort study; RCT, randomized controlled trial; SSB, sugar-sweetened beverages; U, urban; UMIC, upper-middle-income country.

The specific exposures of unhealthy foods or beverages examined in each study are listed in Table 3 and Table S3 in the Supporting Information online. Nineteen studies examined unhealthy beverages, 28 studies (29 articles) examined unhealthy foods and, among the 37 studies, 10 studies reported the effects of both unhealthy foods and beverages, either analyzed separately or combined.^45^^,^^47^^,^^55^^,^^57^^,^^58^^,^^87–90^^,^^92^  Table S3 in the Supporting Information online presents the results disaggregated by categories of exposure (SSB consumption and unhealthy food consumption). Consumption of unhealthy foods and beverages was assessed predominantly via food frequency questionnaires followed by diet diaries, 24-hour dietary recall, and interviews with caregivers.

Assessment of dental caries across the studies was reported using a variety of indices, with some studies reporting dental caries at a single time point, without controlling for baseline dental health, whereas others explored change from baseline to follow-up. The most commonly reported index of dental caries at an individual level was the decayed missing and filled surfaces/teeth (dmfs/dmft) Index (n = 24). However, the reported outcomes varied substantially across studies (eg, presence vs absence, single score, change in index score, or change in a single tooth indicator, eg, first molar). Other studies utilized the International Caries Detection and Assessment System (ICDAS) (n = 1), a combination of dmfs/dmft and ICDAS (n = 3), a combination of the WHO classification and dmft (n = 1), while others relied on parental reports of “dental problems” (cavities, fillings, or extractions) (n = 2), visual and tactile dental examinations without reference to a classification or index (n = 3), study bespoke indices (n = 2), or did not specify (n = 1) (Table 3).

### Participants characteristics

The baseline age of participants ranged from birth^59^ to 8 years.^64^ Over half of the studies (n = 20) included children aged < 2 years at baseline. The oldest ages at follow-up were 9 years^64^ and 18 years.^82^

### Risk-of-bias assessment

The risk-of-bias assessment for individual studies and each domain is presented in Figure S1 in the Supporting Information online. Of the 37 studies, none had an overall low risk of bias (Figure S1 in the Supporting Information online). Five studies (13.5%) had a moderate overall risk of bias,^53^^,^^67^^,^^68^^,^^82^^,^^83^ 25 studies (67.6%) had an overall serious risk of bias,^44^^,^^45^^,^^47–52^^,^^55^^,^^58^^,^^59^^,^^63–65^^,^^73^^,^^76–79^^,^^85^^,^^87^^,^^88^^,^^91–93^ and 6 studies (16.2%) were classed as having critical risk of bias.^57^^,^^66^^,^^80^^,^^89^^,^^90^^,^^94^ One study (2.7%)^46^ was classed as having “no information” for Domain 2 (bias due to selection of participants). Therefore, an overall assessment of “no information” was given. Figure S2 in the Supporting Information online summarizes the main biases identified across studies.

### Synthesis of results

Thirty-seven studies reported the effects of unhealthy foods and beverage consumption on child dental caries experience (Table 3).

In total, 17 studies considered unhealthy beverage consumption. These covered a variety of types of SSBs, including sodas, fruit-flavored drinks, cordials, powdered sweet drinks, juice with added sugar, and caffeinated drinks. No studies examined artificially sweetened beverages (ASBs) or 100% fruit juice alone. With the exception of 3 studies,^67^^,^^77^^,^^79^ all found an association between SSB consumption and dental caries.

Twenty-two studies examined unhealthy food exposures. Almost all studies examined foods high in sugar consumption, namely sweets/candy/confectionary. One study included takeaways and instant noodles as well as sweet foods,^90^ and another study included “high-fat foods” (meat pie, hamburger, hot dog, sausage or sausage roll, chips or French fries, potato chips, biscuits, doughnuts, cake, and chocolate) as well as SSBs.^58^ The results of the individual studies are presented in Table S3 in the Supporting Information online, which provides the summary statistics, effect estimates, and confidence intervals for all included studies. The 6 studies that were judged as being at critical risk of bias were not reported on in the following narrative synthesis,^57^^,^^66^^,^^80^^,^^89^^,^^90^^,^^94^ in line with guidance.^96^

Only 2 studies did not report a significant association between unhealthy food consumption and dental caries.^67^^,^^68^

#### Consumption of unhealthy beverages and dental caries

Ten studies examined unhealthy beverage consumption of children aged <2 years; all were assessed as being at serious risk of bias. Five of the 10 studies reported significant positive associations between SSB consumption and risk of dental caries (Table S3 in the Supporting Information online).^47^^,^^48^^,^^73^^,^^92^^,^^93^

One study recorded intake of SSBs for children using 3-day diet diaries analyzed at age 1 year, 2 years, 3 years, 4 years, and 5 years and cumulatively through ages 1 year–5 years (n = 396).^73^ A dental examination was conducted between the ages of 4 years and 7 years to visually check for the presence or absence of dental caries. Consumption of sugar-containing soda pop and powdered beverages, and, to a lesser extent, 100% juice was associated with increased caries risk.^73^ Logistic regression analysis to predict overall caries experience at age 4 years–7 years, from combined 1 year–5 years exposure variables, found significant associations for soda pop (OR: 2.2 [1.4, 3.6] for high intake vs none or low intakes; *P* < .05) and for sugar-containing beverages made from powder (OR: 2.0 [1.2, 3.4] for high intake vs none or low intakes; *P* < .05).^73^ Similar data were presented by Wigen et al (2015)^93^ in their multivariable regression analysis controlling for family characteristics and oral health behaviour at age 1.5 years and 5 years. Consumption of at least 1 sugary drink per week at age 1.5 years increased the risk of caries experience at 5 years of age (OR: 1.8 [1.1, 2.9]), compared with children being offered sugary drinks less than once a week.^93^

Two further studies grouped their analysis of SSBs and sugary foods and reported a positive association overall with risk of dental caries.^52^^,^^54^ One of these studies investigated the risk of dental caries in permanent (adult) molar teeth in relation to the total number of sugar items introduced to the child before age 6 months. The authors combined SSBs and sugary foods in a multivariate analysis and reported that, for each 1-item increase in sweet food consumption at age 6 months, there was an association with permanent molar caries (aOR: 1.27 [1.02, 1.59]).^54^

One study reported no significant associations between intake of SSBs at 10 months–12 months and caries at 6 years.^79^ A further study reported mixed findings between Early Childhood Caries (ECC) and nonwater beverage consumption, with a positive association between juice drink intake and incidence of caries (OR: 2.0 [1.0, 4.2] [data as reported]) but no association with intake speed (intermittent vs rapid intake), use of a bottle, or intake at night time.^63^

In children aged 2 years to <5 years, 6 studies examined SSB consumption; all were assessed as being at serious risk of bias. Three of the 6 studies reported significantly greater odds of caries with greater SSB consumption.^45^^,^^55^^,^^88^ Pang et al (2015)^45^ followed up 3–6-year-old children at baseline for 2 years (n = 887), with parents completing a questionnaire to establish frequency/day of sugary beverages. New caries was reported using the decayed, filled, and missing teeth (dfmt) index, with number of decayed teeth, missing teeth due to caries, and filled teeth due to caries reported individually. Significantly greater odds of caries with greater SSB consumption was reported (OR: 3.73 [1.55, 8.97]). Skafida et al (2018)^88^ had a similar study design, utilizing a food frequency questionnaire to determine SSB intake/month and the decayed, extracted, and filled teeth (deft) index to assess for dental caries. Children were aged 2 years at baseline and followed up for 3 years, with a greater odds of caries with greater SSB consumption (OR: 1.26 [1.01, 1.55]; *P* < .05). Grindefjord et al (1996)^55^ examined children aged 2.5 years for 12 months and the effects of consumption of sugar-containing beverages on odds of caries (>2/d vs <2/d OR: 1.79 [1.00, 3.15]; *P* = .045).

One further study of children aged 3 years–4 years at baseline followed for 2 years reported no association between consumption of carbonated beverages and caries (dmft) in univariable analyses (1 time/d: B = 0.21 [−0.71, 1.14]; *P* = .651; >1 time/d: B = 0.45 [−0.89, 1.79]; compared with <1 time/d),^77^ but did report an association between dental caries (dmft) and consumption of sugary drinks/snacks at night in multivariable analysis (sometimes: B = 0.88 [0.20, 1.56]; *P* = .011; always: B = 1.19 [0.13, 2.25]; *P* = .028). A study by Hooley et al (2012)^58^ assessed intake of high-fat foods (including chocolate, doughnuts, cake, etc., which often contain large amounts of sugar) and intake of sugary beverages (fruit juice, soft drink, or cordial [nondiet] consumed 24 h prior to the survey). “Dental problems” (caries) were self-reported by parents. Children were aged 4 years–5 years at baseline (“wave 1”), with follow-up at 6 years–7 years (“wave 2”) and 8 years–9 years (“wave 3”). In a prospective logistic regression analysis, the odds of “dental problems” at wave 2 (aOR: 1.10 [linearized standard error {LSE}: 0.04]; *P* = .02) and wave 3 (aOR: 1.13 [LSE: 0.06)]; *P* = .01) increased significantly with high-fat food consumption at the preceding wave, and with sugary beverage consumption at wave 3 (aOR: 1.10 [LSE: 0.04]; *P* = .01). No significant association was found at wave 2 for sugary beverage consumption and “dental problems” (aOR: 1.02 [LSE: 0.03]; *P* = .56).^58^ Ismail et al (2008)^59^ explored children’s sugary beverage (“soda”) intake across 1 week through a questionnaire, with those diagnosed with ECC and Severe Early Childhood Caries (S-ECC) reporting only a significant association for soda intake and S-ECC (OR ± standard deviation 1.25 ± 0.13 [*P* = .04]).

One study assessed SSB intake (frequency/wk) and dental caries outcomes among 494 children aged 8 years–9 years at baseline with a 1-year follow-up. A significant association between children “often” rather than “seldom” consuming homemade (aOR: 1.7 [1.1, 2.9]; *P* < .05 [data as reported]) and carbonated sugary beverages (aOR: 1.9 [1.0, 3.7]; *P* < .01 [data as reported]) and dental caries was reported.^64^ One further study combined analysis of SSB intake along with 3 sugary food categories (1. cake, biscuits and cookies, 2. chocolate and 3. sweet desserts) and did not report a significant association with dental caries in the first permanent molar teeth (incidence rate ratio [IRR]: 0.96 [0.73, 1.27]; *P* = .80).^67^

#### Consumption of unhealthy food items and dental caries

Twenty-two studies examined unhealthy food consumption and dental caries (Table S3 in the Supporting Information online). Among the 11 studies (12 articles) involving children of < 2 years at baseline, 10 studies (11 articles) reported a significant positive association between the consumption of unhealthy food, namely sweets and other sugary snacks, and caries experience in children.^47^^,^^49^^,^^51^^,^^53^^,^^54^^,^^65^^,^^75^^,^^78^^,^^82^^,^^87^^,^^92^ Nine of the 12 articles were at serious risk of bias, with the remainder assessed to be at moderate risk of bias.

The impact of increasing sugar intake on the risk of dental caries experience was also reported.^54^^,^^65^ One study found a significant relationship between increasing sweet food consumption as reported at age 6 months and dental caries in the first permanent molar teeth (aOR: 1.27 [1.02, 1.59]; *P* = .036).^54^ Lopes-Gomes et al (2021)^65^ investigated children’s mean daily intake of snacks containing sugar between main meals and caries incidence. The study reported that children who increased their daily sugar intake (relative risk [RR]: 1.67 [1.09–2.52]) and those who maintained a high sugar intake (RR: 1.81; [1.14–2.87]) had a greater risk of caries into dentine.

Two of the 10 studies reported mixed findings. Peres et al (2016)^82^ reported significantly higher dental caries prevalence and mean DMFT score among high sugar consumers (IRR: 1.67 [1.23, 2.25]) but not among those with increasing sugar consumption from baseline to endline (IRR: 1.22 [0.94, 1.59]) (moderate risk of bias). Chaffee et al (2015)^49^ reported significant differences in early childhood caries in the high vs low tertile for a “sweet index” at age 12 months, but not at age 6 months (serious risk of bias).

One study did not find an association between sugary foods trajectories (“medium” and “highest”) and ECC (medium: IRR: 1.30 [0.85, 2.0]; *P* = .228; highest: IRR: 0.90 [0.47, 1.70]; *P* = .019; overall *P* = .737).^68^

Of the 22 studies examining unhealthy food consumption, 10 assessed children aged 2 years to <5 years. Nine studies reported a significant positive association between unhealthy foods and dental caries (1 moderate risk of bias, 8 serious risk of bias).^44–46^^,^^50^^,^^55^^,^^58^^,^^83^^,^^85^^,^^88^ One study, involving 3-year-old children attending nurseries from low socioeconomic backgrounds, compared daily frequency and amount of sugar intake while in nursery, while at home, and overall intake with caries risk.^83^ Sugar intake was based upon a weighed inventory method, in which all sugary food and drink consumed during 2 separate 3 nonconsecutive days was independently weighed and analyzed using food composition tables. Children with the highest frequency of sugar consumption (4 ± 5 times per d) at nursery were 4.7 times more likely to have a high caries increment over 1 year, compared with those with the lowest frequency (1 ± 2.9 times per day) (OR: 4.70 [2.70 ± 8.18]; *P* = .001). Children having more than 32.6 g of sugar daily at nursery were 2.99 times more likely to have a high caries increment than those having less than that amount (OR: 2.99 [1.82 ± 4.91]; *P* = .001). Those children with the highest overall sugar intake (5 or more times per d) were 6.29 times more likely to have a high caries increment (OR: 6.29 [2.56 ± 15.45]). Similarly, the study by Skafida et al (2018)^88^ reported that frequent consumption of sugar-rich foods (soft drinks, sweets, chocolate, and yoghurts) was associated with dental decay in children under 5 years.^88^ Interestingly, children who did consume sweets or chocolate more frequently (once/d or more) but brushed their teeth more often (once/d; twice/d or more) reduced their chance of developing caries, respectively (OR: 2.11 [1.28, 3.49]; 2.26 [1.63, 3.15]), compared with children brushing their teeth less than once/day (OR 3.60 [1.11, 11.68]).^88^ Overall, the authors found an incremental association between decreasing frequency of toothbrushing at age 2 years and increased risk of dental caries at age 5 years (OR 1.39–2.17).^88^

One further study of children aged 3 years–4 years at baseline followed for 2 years reported no overall association between consumption of sweet snacks and dmft (1 time/d: B = 0.21 [−0.71, 1.14]; *P* = .651; >1 time/d: B = 0.45 [−0.89, 1.79]; *P* = .510 compared with <1 time/d), but did report an association between early childhood caries and sweet snack consumption of >1 time/day vs <1 time/day (OR = 1.86, [1.06, 3.27]; *P* = .03).^77^

There was only 1 study of unhealthy food consumption and dental caries outcomes among children aged 5 years to ≤10 years at baseline, which reported no significant association (IRR: 0.96 [0.73, 1.27]; *P* = .80).^67^ This analysis combined consumption of sugary snacks and SSBs assessing frequency/day (≥2 vs <2) of intake reported in a 3-day food diary and presence of dental caries in first permanent molar teeth at a 2-year follow-up.

## DISCUSSION

### Summary of evidence

The evidence synthesized in this review pointed toward a positive relationship between consumption of unhealthy beverages, mainly SSBs, and/or unhealthy foods (mainly foods high in free sugars) and dental caries. A slightly greater number of studies examined the effects of unhealthy food consumption (22 studies) than SSB consumption (17 studies) (excluding those with critical risk of bias) on dental caries, with unhealthy foods focusing predominantly on sweets, candy, or confectionary consumption. Most studies reported greater risk of dental caries with greater consumption of unhealthy foods, beverages, or combinations of the two. However, 3 studies reported no associations between exposure to unhealthy foods and dental outcomes.^67^^,^^68^^,^^79^ Effect sizes varied across studies, but given the call for urgent action on the prevention of dental caries, particularly in low- and middle-income countries where oral health services are most limited, risk factors for dental caries are a major public health concern.^6^

In the studies reporting a positive association between sugary foods and dental caries, higher frequency of daily sugary food intake and intake at night by children were also found to be significantly associated with dental cares^65^^,^^78^^,^^83^ or considered to be risk factors.^45^ Consumption of sugary foods at bedtime is reported to increase the risk of caries development, as saliva flow rate reduces, leading to reduced clearance of sugar from the teeth and sustained low pH levels, which favour enamel demineralization.^82^ Sugary intake at bedtime was also reported, in recent systematic reviews, to increase caries risk.^97^^,^^98^

The study by Skafida et al (2018)^88^ reported that children consuming sweets or chocolate more frequently (once/d or more) but brushed teeth more often (once/d; twice/d or more) reduced their chance of developing caries (OR: 2.11 [1.28, 3.49]; 2.26 [1.63, 3.15], respectively) compared with children brushing teeth less than once/day (OR 3.60 [1.11, 11.68]).^88^ This highlights toothbrushing as a confounder among studies investigating sugar intake, but the data nonetheless suggest that prolonged sugar intake and snacking may only be attenuated to a certain extent by toothbrushing alone. Toothbrushing, with regards to frequency or whether the activity was supervised by a caregiver, was considered by studies in this review. Lower frequency, lack of toothbrushing after night time consumption of sugary foods, and unsupervised brushing were reported to increase the risk of dental caries.^45^^,^^47^^,^^83^^,^^88^^,^^92^

Other reported risk factors for dental caries identified in this review are supportive of the wider literature and include socioeconomic deprivation, lower fluoride exposure, and a history of dental caries.^45^^,^^47^^,^^54^^,^^65^^,^^78^^,^^83^^,^^92^ Studies that did not account for such confounding factors were recorded appropriately under Domain 1 of the ROBINS-1 tool.

In studies reporting a significant association between unhealthy beverages and dental caries, the consumption of sugary drinks at night and at increased frequency throughout the day was suggested to increase risk for dental caries, as reported for unhealthy foods.^45^^,^^47^^,^^48^^,^^52^^,^^54^^,^^77^^,^^88^^,^^93^ One study reported that exposure to at least 1 sugary drink per week at 18 months of age (OR = 1.8, CI = 1.1, 2.9), alongside having teeth brushed less than twice daily (OR = 2.1, CI = 1.3, 3.6), was associated with caries experience at age 5 years, after controlling for other oral health behaviors and family characteristics.^93^ The suggestion that introduction of sugar at a young age, such as through SSBs, increases caries risk and may establish an increasing sugar trajectory of consumption throughout childhood, was also reported in other studies.^48^^,^^52^^,^^64^ As a result, there is support for current recommendations to avoid sugars for very young children and interventions to promote healthier, less cariogenic early feeding practices.^48^ WHO Guidelines recommend that intake of free sugars should form <10% of EI for children, with further recommendations to reduce that figure to <5% of EI for caries prevention.^97^^,^^99–101^ Minimizing sugar intake in childhood to minimize lifetime risk of caries is important, given that dental caries develops over time, largely as a result of long-term exposure to this dietary risk factor (free sugars).^101^ Exclusive breastfeeding from birth to 6 months, as recommended by WHO, would also reduce exposure to unhealthy food and beverage consumption in early life.^101^

However, not all studies reported consistent associations. Three studies reported no significant association (moderate risk of bias^67^^,^^68^ and serious risk of bias^79^), and 2 studies reported mixed findings (serious risk of bias).^58^^,^^59^ Mahboobi et al (2021)^67^ was the only study to explore children of >5 years at baseline, and they did not find an association between caries incidence and intake of 2 or more sugary snacks/day, or an association with oral hygiene status. The high drop-out rate (41.6%) from baseline (7–8-year-old children) to follow-up (9–10-year-old children), possible under-reporting of sugary snack intake by caregivers, as well as limited caries assessment (first permanent molar teeth only) could help account for the lack of association. A bespoke caries index, Caries Assessment Spectrum and Treatment (CAST), was used by the authors, which impairs comparison with other studies utilizing more widely used and accepted indices (eg, dfmt). Moreover, Hooley et al (2012)^58^ did not find a significant increased risk of odds of dental caries for children aged 6 years–7 years with high sugary beverage intake at ages 4 years–5 years (aOR: 1.02 [LSE: 0.03]; *P* = .56). However, the authors did report an increased risk of dental caries for children aged 8 years–9 years based on sugary beverage consumption at 6 years–7 years (aOR: 1.10 [LSE: 0.04]; *P* = .01) as well as significantly greater odds of dental caries at ages 6 years–7 years and 8 years–9 years, respectively, based on previous high-fat food intake (aOR: 1.10 [LSE]: 0.04]; *P* = .02); (aOR: 1.13 [LSE: 0.06)]; *P* = .01). The study relied on caregivers to report dental caries and SSB consumption. Therefore, under-reporting of dental caries is probable, combined with potential recall bias in the dietary recall and potential lack of a representative unhealthy food and beverage intake, with reporting limited to a period of 24 hours.^58^

Temporal changes in consumption of unhealthy foods and beverages will have taken place over the period under consideration in this review, varying within and between countries. The estimated SSB consumption among US children and adolescents (2–19 years) increased steadily from 1965, peaked in the year 2000, then declined.^102^ In other high- and middle-income countries, however, SSB intakes for children aged 1 year–11 years have remained relatively stable from 1990 to 2015, for example, in Australia, Canada, China, South Korea, and the UK.^102^ National intake trends also vary by socioeconomic status, with declines sometimes evident among higher socioeconomic groups, while remaining stable or increasing among lower socioeconomic groups (eg, Morgan et al 2021^103^). In low-income countries, quantitative data on the consumption of SSBs and ultraprocessed foods in children remain sparse,^30^^,^^104^ but are likely to show upward trends with increased availability and affordability of highly processed foods.

### Strengths and limitations

Strengths of the review are the inclusion of studies dating from 1971, with no restrictions on language or country. A recent systematic review assessing the relationship between dietary free sugars and dental caries in children was limited to English language only.^97^ The focus of this review on children aged ≤10 years added valuable insights for this less-extensively studied age group. The study selection included longitudinal study designs to gain stronger evidence of association, rather than relying on evidence from cross-sectional study designs. A criterion of unhealthy food and beverages was agreed upon with WHO technical advisors for this review, with the importance of such a standardized approach recommended in a previous systematic review.^97^ In the absence of a universal definition of unhealthy foods and beverages, a food-based and nutrient-based approach was employed to ensure a comprehensive identification of relevant exposures. While the term “unhealthy food and beverages” is used, alternative terms such as “foods of high energy and minimal nutritional value,” “highly processed foods” or “foods high in salt” could also be used.

Some limitations are worthy of consideration. A major limitation of the evidence was the high heterogeneity across studies in the reporting of outcomes and exposures. Dietary exposures and categories of consumption were rarely predefined in studies, and tended to be identified at the analysis stage. Other reviews have similarly highlighted the need for further well-designed longitudinal studies and standardized study protocols.^97^^,^^105^ RCTs on unhealthy food consumption would be unethical, as it would require giving children unhealthy foods. The heterogeneity across the included studies arose from the indices used to measure caries outcomes (dfmt, deft, CAST index, ICDAS, etc.), the data collection tools, the recall periods, and the categorization used in dietary analysis (exposures) as well as from the variety in the study populations. These issues, along with other sources of heterogeneity, precluded meta-analysis of the data.

Self-reports of food and drink intake are also subject to recall bias, social desirability, and the effects of repeated assessments. Retrospective recall periods of dietary intake varied from 1 day to 7 days, mostly completed by the parent/guardian, but sometimes by a member of the research team. Attrition of samples over time and the effects of missing data were considered in the risk of bias assessment, but are also likely to have contributed to residual confounding.

## CONCLUSIONS

The consumption of unhealthy food and beverages in children of ≤10 years appears to increase the risk of dental caries. Further longitudinal studies with high-quality dietary assessments, especially including low-income countries and children aged >5 years at baseline, are recommended to develop a more robust evidence base to assist in the development of policy recommendations. Standardized criteria for unhealthy food and beverages should be used, as well as appropriate caries assessment by oral health professionals using accepted caries indices. Baseline caries assessment and consideration of confounders is fundamental, with appropriate follow-up periods, to assess caries, given that caries development occurs over time.

## Supplementary Material

nuad147_Supplementary_Data

## Supporting Information

The following Supporting Information is available through the online version of this article at the publisher’s website.

*Table S1* **Database searches for the effects of unhealthy food and beverage consumption among children aged ≤10 years on risk of dental caries**

*Table S2*  **Assessment criteria for overall risk of bias for nonrandomized studies of interventions**

*Table S3*  **Synthesis of the results of studies on the effect of unhealthy food and beverage consumption on dental caries outcomes in children aged ≤10 years**

*Figure S1* **Individual risk-of-bias assessment for nonrandomized studies reporting the effect of unhealthy food and beverage consumption on dental caries in children aged ≤10 years, using ROBINS-I**

*Figure S2*  **Summary risk of bias across the included studies on the effect of unhealthy food and beverage consumption on dental caries in children aged ≤10 years**
