# Supplementary material for: Impact of unhealthy food and beverage consumption on children’s risk of dental caries: a systematic review
Source: Nutr Rev. 2023 Dec 12;82(11):1539–55. doi: 10.1093/nutrit/nuad147 (PMC11465133; doi:10.1093/nutrit/nuad147)
Supplement: nuad147_Supplementary_Data [file nuad147_supplementary_data.zip › nuad147_Supplementary_Data/Supplementary Materials R2 EKR.docx]

**Impact of unhealthy food and beverage consumption in children on risk of dental caries: a systematic review**

*Jessica F Large, Claire Madigan, Rebecca Pradeilles, Oonagh Markey, Benjamin Boxer, Emily K Rousham*

**Supplementary Tables and Figures**

**Table S1** Database searches for the effects of unhealthy food and beverage consumption among children aged ≤10 years on risk of dental caries

**Search Name: Cochrane searches**

ID Search Hits

#1 (infant):ti,ab,kw 49857

#2 (infants):ti,ab,kw 32907

#3 (infancy):ti,ab,kw 3763

#4 MeSH descriptor: [Infant] this term only 21560

#5 (toddler*):ti,ab,kw 1748

#6 (baby):ti,ab,kw 5083

#7 (babies):ti,ab,kw 4875

#8 MeSH descriptor: [Child] this term only 48734

#9 ("Child"):ti,ab,kw 146976

#10 (school child*):ti,ab,kw 20996

#11 (boy):ti,ab,kw 711

#12 (boys):ti,ab,kw 6378

#13 (girl):ti,ab,kw 1470

#14 (girls):ti,ab,kw 7051

#15 ("pre-school*"):ti,ab,kw 614

#16 ("kindergar*"):ti,ab,kw 0

#17 ("elementary school"):ti,ab,kw 1021

#18 (primary school):ti,ab,kw 9832

#19 #1 OR # 2 OR #5 OR #6 OR #7 OR #8 OR #9 OR #10 OR #11 OR #13 OR #15 OR #16 OR #17 OR #18 1068829

#20 MeSH descriptor: [Snacks] explode all trees 282

#21 (snack):ti,ab,kw 1519

#22 #20 OR #21 1611

#23 MeSH descriptor: [Candy] explode all trees 792

#24 (candy):ti,ab,kw 369

#25 (candies):ti,ab,kw 64

#26 (sweets):ti,ab,kw 293

#27 (confection*):ti,ab,kw 103

#28 (sweet food):ti,ab,kw 525

#29 MeSH descriptor: [Chocolate] explode all trees 60

#30 (chocolat*):ti,ab,kw 1077

#31 (salt*):ti,ab,kw 7071

#32 (salty food):ti,ab,kw 125

#33 (savoury):ti,ab,kw 158

#34 #23 OR #24 OR #25 OR #26 OR #27 OR #28 OR #29 OR #30 OR #31 OR #32 OR #33 9795

#35 MeSH descriptor: [Fast Foods] explode all trees 105

#36 ("fast-food"):ti,ab,kw 601

#37 (street food):ti,ab,kw 29

#38 (junk food*):ti,ab,kw 77

#39 (convenience food*):ti,ab,kw 264

#40 (ready-prepared food*):ti,ab,kw 2

#41 (ready to eat meal*):ti,ab,kw 58

#42 (takeaway food*):ti,ab,kw 23

#43 (take-away food*):ti,ab,kw 17

#44 (takeout food*):ti,ab,kw 5

#45 (take-out food*):ti,ab,kw 8

#46 (fried food*):ti,ab,kw 158

#47 (ultra-processed food*):ti,ab,kw 32

#48 (ultraprocessed food*):ti,ab,kw 36

#49 (processed food*):ti,ab,kw 650

#50 (processed meat):ti,ab,kw 205

#51 (fatty food*):ti,ab,kw 3259

#52 #35 OR #36 OR #37 OR #38 OR #39 OR #40 OR #41 OR #42 OR #43 OR #44 OR #45 OR #46 OR #47 OR #48 OR #49 OR #50 OR #51 4984

#53 MeSH descriptor: [Dietary Sucrose] explode all trees 305

#54 (sugar):ti,ab,kw 8085

#55 (sugars):ti,ab,kw 961

#56 (sugary):ti,ab,kw 213

#57 MeSH descriptor: [Sweetening Agents] explode all trees 736

#58 (sweetener*):ti,ab,kw 550

#59 #53 OR #54 OR #55 OR #56 OR #57 OR #58 9425

#60 ("unhealthy"):ti,ab,kw 1513

#61 (inappropriate food*):ti,ab,kw 103

#62 (non-nutritive):ti,ab,kw 300

#63 (nonnutritive):ti,ab,kw 383

#64 (nutrient poor):ti,ab,kw 301

#65 (energy dense food*):ti,ab,kw 463

#66 (less healthy meal*):ti,ab,kw 941

#67 (less healthy food*):ti,ab,kw 1705

#68 (low nutrient):ti,ab,kw 1512

#69 (nutritive value):ti,ab,kw 496

#70 (energy-dense):ti,ab,kw 436

#71 #60 OR #61 OR #62 OR #63 OR #64 OR #65 OR #66 OR #67 OR #68 OR #69 OR #70 6356

#72 (complementary food*):ti,ab,kw 808

#73 (complementary diet):ti,ab,kw 644

#74 (complementary meal*):ti,ab,kw 191

#75 #72 OR #73 OR #74 1175

#76 MeSH descriptor: [Beverages] explode all trees 6029

#77 (beverage*):ti,ab,kw 6631

#78 (soda):ti,ab,kw 413

#79 (sodas):ti,ab,kw 47

#80 (carbonated drink):ti,ab,kw 147

#81 (sweet drink*):ti,ab,kw 172

#82 (sweetened drink*):ti,ab,kw 407

#83 (soft drink*):ti,ab,kw 559

#84 #76 OR #77 OR #78 OR #79 OR #80 OR #81 OR #82 OR #83 11157

#85 #22 OR #34 OR #52 OR #59 OR #71 OR #75 OR #84 37956

#86 ("intake"):ti,ab,kw 54242

#87 (intakes):ti,ab,kw 4651

#88 (consum*):ti,ab,kw 70753

#89 (feeding):ti,ab,kw 22133

#90 (eating):ti,ab,kw 14676

#91 (drinking):ti,ab,kw 11635

#92 (eat):ti,ab,kw 3672

#93 (drink):ti,ab,kw 6818

#94 #86 OR #87 OR #88 OR #89 OR #90 OR #91 OR #92 OR #93 139621

#95 MeSH descriptor: [Animals] this term only 9792

#96 MeSH descriptor: [Humans] this term only 591274

#97 (#19 AND #85 and #94) NOT (#95 NOT #96) 16629

#98 ("randomized-controlled trial"):pt 498138

#99 (controlled clinical trial):pt 323841

#100 (randomized):ti,ab,kw 901454

#101 (trial):ti,ab,kw 825752

#102 (groups):ti,ab,kw 475952

#103 (comparative study):pt 166014

#104 MeSH descriptor: [Control Groups] explode all trees 111

#105 MeSH descriptor: [Follow-Up Studies] explode all trees 59090

#106 (follow-up stud*):ti,ab,kw 219260

#107 (follow-up assessment):ti,ab,kw 58334

#108 MeSH descriptor: [Prospective Studies] explode all trees 91786

#109 (prospective stud*):ti,ab,kw 206021

#110 MeSH descriptor: [Evaluation Studies as Topic] explode all trees 50832

#111 (evaluat*):ti,ab,kw 492538

#112 (quasi experiment*):ti,ab,kw 5115

#113 (quasiexperiment*):ti,ab,kw 4452

#114 MeSH descriptor: [Interrupted Time Series Analysis] explode all trees 51

#115 (ITS stud*):ti,ab,kw 108192

#116 (time series):ti,ab,kw 7967

#117 (time point*):ti,ab,kw 69107

#118 MeSH descriptor: [Controlled Before-After Studies] explode all trees 71

#119 (controlled):ti,ab,kw 744775

#120 (CBA stud*):ti,ab,kw 267

#121 (pre test):ti,ab,kw 26376

#122 (pretest):ti,ab,kw 10268

#123 (post test):ti,ab,kw 41465

#124 (posttest):ti,ab,kw 11946

#125 (pre intervention):ti,ab,kw 36566

#126 (post intervention):ti,ab,kw 61277

#127 (before-after stud*):ti,ab,kw 1459

#128 ("before and after"):ti,ab,kw 59238

#129 (nonrandom):ti,ab,kw 283

#130 (non-random*):ti,ab,kw 5626

#131 MeSH descriptor: [Cohort Studies] explode all trees 148452

#132 (cohort stud*):ti,ab,kw 49549

#133 (longitudinal stud*):ti,ab,kw 18887

#134 #98 OR #99 OR #100 OR #101 OR #102 OR #103 OR #104 OR #105 OR #106 OR #107 OR #108 OR #109 OR #110 OR #111 OR #112 OR #113 OR #114 OR #115 OR #116 OR #117 OR #118 OR #119 OR #120 OR #121 OR #122 OR #123 OR #124 OR #125 OR #126 OR #127 OR #128 OR #129 OR #130 OR #131 OR #132 OR #133 1418750

#135 #97 AND #134 with Publication Year from 1971 to 2020, with Cochrane Library publication date Between Jan 1971 and Dec 2020, in Trials 15393

#136 ("editorial"):pt 2752

#137 (comment):pt 1882

#138 (news):pt 334

#139 ("Letter"):pt 12061

#140 (review):pt 17935

#141 ("systematic review"):pt 59

#142 ("meta-analysis"):pt 530

#143 ("meta-analysis"):ti,ab,kw 17978

#144 ("meta analyses"):ti,ab,kw 17978

#145 (retracted publication):pt 485

#146 (retraction of publication):pt 58

#147 (retraction of publication):ti,ab,kw 42

#148 (retraction of publication):pt 58

#149 #136 OR #137 OR #138 OR #139 OR #140 OR #141 OR #142 OR #143 OR #144 OR #145 OR #146 OR #147 OR #148 48859

#150 #135 NOT #149 with Cochrane Library publication date Between Jan 1971 and Dec 2020 15224

**Database: EMBASE**

| Search | Query | Results |
| --- | --- | --- |
| #12 | #10 not #11 | 6982 |
| #11 | Limit #10 to conference abstracts | 2414 |
| #10 | #8 NOT #9 | 9396 |
| #9 | Search: editorial.pt OR comment.pt OR news.pt OR letter.pt OR review.pt OR "systematic review".pt OR "systematic review".tw OR "meta-analysis".pt OR "meta-analysis".tw OR "meta-analyses".tw OR "retracted publication".pt OR "retraction of publication".pt OR "retraction of publication".tw OR "retraction notice".tw | 4726267 |
| #8 | Search: #6 and #7 | 10108 |
| #7 | Search: randomized controlled trial.pt OR controlled clinical trial.pt OR randomized.tw OR trial.tw OR groups.tw OR comparative study.pt OR control groups.mp OR follow-up studies.mp OR follow-up stud*.tw OR follow-up assessment.tw OR prospective studies.mp OR prospective stud*.tw OR "evaluation studies as topic".mp OR evaluat*.tw OR quasi experiment*.tw OR quasiexperiment*.tw OR interrupted time series analysis.mp OR ITS stud*.tw OR time series.tw OR time point*.tw OR controlled before-after studies.mp OR controlled.tw OR CBA stud*.tw OR pre test.tw OR pretest.tw OR post test.tw OR posttest.tw OR pre intervention.tw OR post intervention.tw OR before-after stud*.tw OR "before and after".tw OR nonrandom*.tw OR non-random*.tw OR cohort studies.mp OR cohort stud*.tw OR longitudinal stud*.tw | 9040729 |
| #6 | Search: #4 NOT #5 | 21571 |
| #5 | Search: (animals.mp NOT humans.mp) | 767206 |
| #4 | 1 and 2 and 3 | 21724 |
| #3 | Search: intake.tw OR intakes.tw OR consum*.tw OR feeding.tw OR eating.tw OR drinking.tw OR eat.tw OR drink.tw | 1281552 |
| #2 | Search: beverages.mp OR beverage*.tw OR soda.tw OR sodas.tw OR carbonated drink*.tw OR sweet drink*.tw OR sweetened drink*.tw OR soft drink*.tw OR complementary food*.tw OR complementary diet.tw OR complementary meal*.tw OR unhealthy.tw OR inappropriate food.tw OR inappropriate foods.tw OR non-nutritive.tw OR nonnutritive.tw OR nutrient poor.tw OR less healthy meal*.tw OR less healthy food*.tw OR low nutrient.tw OR nutritive value.tw OR energy-dense.tw OR dietary sucrose.mp OR sugar.tw OR sugars.tw OR sugary.tw OR sweetening agents.mp OR sweetener.tw OR sweeteners.tw OR fast foods.mp OR fast food*.tw OR street food*.tw OR junk food*.tw OR convenience food*.tw OR ready-prepared food*.tw OR ready to eat meal*.tw OR takeaway food*.tw OR take-away food*.tw OR takeout food*.tw OR take-out food*.tw OR fried food*.tw OR ultra-processed food*.tw OR ultraprocessed food*.tw OR processed food*.tw OR processed meat*.tw OR fatty food*.tw OR candy.mp OR candy.tw OR candies.tw OR sweets.tw OR confection*.tw OR sweet food*.tw OR chocolate.mp OR chocolat*.tw OR salt*.tw OR savoury.tw OR Snacks.mp OR snack*.tw | 450238 |
| #1 | Search: infant.mp OR infant.tw OR infants.tw OR infancy.tw OR toddler*.tw OR baby.tw OR babies.tw OR child.mp OR child*.tw OR schoolchild*.tw OR boy.tw OR boys.tw OR girl.tw OR girls.tw OR pre school*.tw OR kindergar*.tw OR elementary school*.tw OR primary school*.tw | 3169372 |

**Database: PubMed**

| Search | Query | Results |
| --- | --- | --- |
| #16 | #14 NOT #15 | 13,060 |
| #15 | Search: editorial[Publication Type] OR comment[Publication Type] OR news[Publication Type] OR letter[Publication Type] OR review[Publication Type] OR "systematic review"[Publication Type] OR "systematic review"[tiab] OR "meta-analysis"[Publication Type] OR "meta-analysis"[tiab] OR "meta-analyses"[tiab] OR "retracted publication"[Publication Type] OR "retraction of publication"[Publication Type] OR "retraction of publication"[tiab] OR "retraction notice"[tiab] Filters: from 1971/1/1 - 2020/11/27 | 4,922,701 |
| #14 | Search: #13 AND #14 Filters: from 1971/1/1 - 2020/11/30 Sort by: Publication Date | 14,355 |
| #13 | Search: #11 AND #12 | 14,380 |
| #12 | Search: randomized controlled trial[pt] OR controlled clinical trial[pt] OR randomized[tiab] OR trial[tiab] OR groups[tiab] OR comparative study[pt] OR control groups[mh] OR follow-up studies[mh] OR follow-up stud*[tiab] OR follow-up assessment[tiab] OR prospective studies[mh] OR prospective stud*[tiab] OR "evaluation studies as topic"[mh] OR evaluat*[tiab] OR quasi experiment*[tiab] OR quasiexperiment*[tiab] OR interrupted time series analysis[mh] OR ITS stud*[tiab] OR time series[tiab] OR time point*[tiab] OR controlled before-after studies[mh] OR controlled[tiab] OR CBA stud*[tiab] OR pre test[tiab] OR pretest[tiab] OR post test[tiab] OR posttest[tiab] OR pre intervention[tiab] OR post intervention[tiab] OR before-after stud*[tiab] OR "before and after"[tiab] OR nonrandom*[tiab] OR non-random*[tiab] OR cohort studies[mh] OR cohort stud*[tiab] OR longitudinal stud*[tiab] | 9,144,658 |
| #11 | Search: (#1 AND #9 AND #10) NOT (animals[mh] NOT humans[mh]) | 26,811 |
| #10 | Search: intake[tiab] OR intakes[tiab] OR consum*[tiab] OR feeding[tiab] OR eating[tiab] OR drinking[tiab] OR eat[tiab] OR drink[tiab] | 1,008,423 |
| #9 | Search: #2 OR #3 OR #4 OR #5 OR #6 OR #7 OR #8 | 523,824 |
| #8 | Search: beverages[mh] OR beverage*[tiab] OR soda[tiab] OR sodas[tiab] OR carbonated drink*[tiab] OR sweet drink*[tiab] OR sweetened drink*[tiab] OR soft drink*[tiab] | 162,087 |
| #7 | Search: complementary food*[tiab] OR complementary diet[tiab] OR complementary meal*[tiab] | 1486 |
| #6 | Search: unhealthy[tiab] OR inappropriate food[tiab] OR inappropriate foods[tiab] OR non-nutritive[tiab] OR nonnutritive[tiab] OR nutrient poor[tiab] OR less healthy meal*[tiab] OR less healthy food*[tiab] OR low nutrient[tiab] OR nutritive value[tiab] OR energy-dense[tiab] | 23,532 |
| #5 | Search: dietary sucrose[mh] OR sugar[tiab] OR sugars[tiab] OR sugary[tiab] OR sweetening agents[mh] OR sweetener[tiab] OR sweeteners[tiab] | [133,115](https://pubmed.ncbi.nlm.nih.gov/?term=dietary+sucrose%5Bmh%5D+OR+sugar%5Btiab%5D+OR+sugars%5Btiab%5D+OR+sugary%5Btiab%5D+OR+sweetening+agents%5Bmh%5D+OR+sweetener%5Btiab%5D+OR+sweeteners%5Btiab%5D&sort=relevance&size=200&ac=no) |
| #4 | Search: fast foods[mh] OR fast food*[tiab] OR street food*[tiab] OR junk food*[tiab] OR convenience food*[tiab] OR ready-prepared food*[tiab] OR ready to eat meal*[tiab] OR takeaway food*[tiab] OR take-away food*[tiab] OR takeout food*[tiab] OR take-out food*[tiab] OR fried food*[tiab] OR ultra-processed food*[tiab] OR ultraprocessed food*[tiab] OR processed food*[tiab] OR processed meat*[tiab] OR fatty food*[tiab] | 12,955 |
| #3 | Search: candy[mh] OR candy[tiab] OR candies[tiab] OR sweets[tiab] OR confection*[tiab] OR sweet food*[tiab] OR chocolate[mh] OR chocolat*[tiab] OR salt*[tiab] OR savoury[tiab] | 215,735 |
| #2 | Search: Snacks[mh] OR snack*[tiab] | 8,453 |
| #1 | Search: infant[mh] OR infant[tiab] OR infants[tiab] OR infancy[tiab] OR toddler*[tiab] OR baby[tiab] OR babies[tiab] OR child[mh] OR child*[tiab] OR schoolchild*[tiab] OR boy[tiab] OR boys[tiab] OR girl[tiab] OR girls[tiab] OR pre school*[tiab] OR kindergar*[tiab] OR elementary school*[tiab] OR primary school*[tiab] | 3,071,122 |

**Table S2** Assessment criteria for overall risk of bias for non-randomized studies of interventions

| **Overall risk of bias assessment for ROBINS-I*** | **Criteria** |
| --- | --- |
| Low | Study is judged to be at low risk of bias for all domains |
| Moderate | Study is judged to be a low or moderate risk of bias for all domains |
| Serious | Study is judged to be a serious risk of bias in at least one domain-but not at critical risk of bias in any domain |
| Critical | The study is judged to be at critical risk of bias in at least one domain |
| No information | No indication that the study is a serious or critical risk of bias *and* there is a lack of information in one or more key domains of bias |

* From Sterne et al 2016. ^1^

We followed the signaling questions provided in the detailed guidance notes for each domain and also defined the major confounding variables to be considered for the intervention (i.e. exposure to (consumption of) unhealthy foods and beverages) under consideration in included studies (Domain 1). ^1^

**Table S3** Synthesis of results of studies on the effect of unhealthy food and beverage consumption on dental caries outcomes in children aged ≤10 years^†^

| **Study ID** | **Baseline age (mean or range)** | | **Length of follow-up** | | **N^‡^** | | **DAT** | **Exposure** | | **Intake unit** | | **Comparator** | | **Indicator** | | **Outcome** | **Estimate*** | | | **Overall RoB** |
| --- | --- | --- | --- | --- | --- | --- | --- | --- | --- | --- | --- | --- | --- | --- | --- | --- | --- | --- | --- | --- |
| **Exposure: SSB** |  | |  | |  | |  |  | |  | |  | |  | |  |  | | |  |
| 0–<2 y |  | |  | |  | |  |  | |  | |  | |  | |  |  | | |  |
| Anderson 2021 ^2^ | 1 y | | 6 y | | 2400 | | Questionnaire completed by parents with examiner | Sugar-containing beverages | | Frequency/d | | Not consumed vs consumed ≥ 1/day at 1, 2 and 3 y old | | ICDAS | | Dental caries (defs 0 vs >0) at 1, 2, 3, 5 and 7 y. | OR = 1.2 (95% CI = 1.02, 1.60) (1 y), OR = 1.44 (95% CI = 1.05, 1.99) (2 y), OR = 2.01 (95% CI = 1.60, 2.55) (3 y), OR = 1.36 (95% CI = 1.08, 1.80) (5 y), OR = 1.38 (95% CI = 1.09, 1.74) (7 y) | | | Serious |
| Bernabe 2020 ^3^ | 12.8 mo | | 36 mo | | 1111 | | FFQ | Sugar containing beverages | | Frequency/d | | Initial intake (continuous); Deviations from initial intake (continuous) | | dmfs | | Dental caries trajectory | Baseline intake: β = -0.1 95% CI = -0.17, -0.03, P = 0.006; Change in intake: β = -0.14 (95% CI = -0.22, -0.05) P = 0.001 | | | Serious |
| Echeverria 2022 ^4^ | 3-48 mo | | 3 y 9 mo | | 2806 | | Questionnaire completed by caregiver | Sugar-containing beverages | | Trajectory of sugar consumption from 3 to 48 m (always low, always intermediate, growing, and always high) | | Low vs high median sugar consumption | | ICDAS | | Dental caries at 48 mo; cavitated dental caries at 48 mo | Prevalence ratio (adjusted) dental caries = 1.42 (95% CI = 1.17, 1.73); Prevalence ratio cavitated caries = 1.51 (95% CI = 1.19, 1.92), P < 0.001 | | | Serious |
| Jordan 2020 ^5^ | 8-18 mo | | 5 y | | 93 | | FFQ | SSB | | Frequency/d | | Consumed vs. not consumed | | dmfs | | Incident caries dichotomized outcome of caries-free (incidence = 0) vs caries (incidence >0) | OR = 2 (95% CI = 1.0, 4.2) | | | Serious |
| Marshall 2003 ^6,7^ | 6 wk | | 5 y | | 291 | | 3-d diet diaries at 1, 2, 3, 4, 5 y | SSB | | g/d | | Pop/sports drink consumption 12-24 mo; Pop/sports drink and 36-48 mo; Sugar beverages at 12-24 mo | | d_1_ lesions; d_2-3_ lesions | | Caries at 12-36 mo | 12-36 mo: OR = 1.34, P = 0.12; 36-48 mo: OR = 1.33, P = 0.12 | | | Serious |
|  | 1 y | | 3-6 y | | 396 | | 3-d diet diaries at 1, 2, 3, 4, 5 y | SSB | | g/d | | Continuous | | d_1_ lesions; d_2-3_ lesions | | Caries at 4 and 7 y | Soda pop: OR = 2.2 (95% CI = 1.4, 3.6) P < 0.05; Drinks from powder: OR = 2.0 (95% CI = 1.2, 3.4) P < 0.05 | | | Serious |
| Park 2015 ^8^ | 10-12 mo | | 62 mo | | 1269 | | 7-d recall questionnaire | SSB | | Frequency/d | | SSB <1 times/wk vs. none; SSB 1-<3 times/wk vs. none, SSB ≥ 3 times/wk vs. none | | Number of reported caries | | Caries number at 6 y | OR = 1.15 (95% CI = 0.61, 2.18) | | | Serious |
| Sakuma 2007 ^9^ | 1.5 y | | 1.5 y | | 5107 | | FFQ | SSB | | Frequency/d | | Continuous | | Change in caries | | Number of teeth with caries | Four cities/districts: β = 0.34, OR = 1.4 (95% CI = 1.2, 1.7) P < 0.001; β = 0.39, OR = 1.5 (95% CI = 1.3, 1.7) P < 0001; β = 0.19, OR = 1.2 (95% CI = 1.0, 1.4) P < 0.05; Other β = 0.28, OR = 1.3 (95% CI = 1.2, 1.5) P < 0.001 | | | Serious |
| Warren 2009 ^10^ | 6-24 mo | | 18 mo | | 128 | | Questionnaire completed by study coordinator | SSB | | Consumed/not consumed in a week | | Regular consumption vs. not consumed | | Cavitated and non-cavitated dental lesions | | Cavitated (_d2-3_) and non cavitated (_d1_ lesions) | OR = 5.20, (95% CI = 2.0, 13.3) P = 0.001 | | | Serious |
| Watanabe 2014 ^11^ | 1.5 y | | ~21 mo | | 31202 | | FFQ | SSB | | Frequency/d | | Daily consumed vs. not consumed | | Dental caries present or absent | | Dichotomous (0, 1) | OR = 1.56 (95% CI = 1.46, 1.65), P < 0.001 | | | Serious |
| Wigen 2015 ^12^ | 1.5 y | | 3.5 y | | 1095 | | FFQ | SSB | | Frequency/wk | | ≥ once/wk vs.< once/wk | | Sum of dmft | | Dichotomous (0, 1) | OR = 1.9, (95% CI = 1.2, 2.9) | | | Serious |
| 2–<5 y |  | |  | |  | |  |  | |  | |  | |  | |  |  | | |  |
| Grindefjord 1996 ^13^ | 30 mo | | 12 mo | | 692 | | Questionnaire completed by parents at 1, 2.5 and 3.5 y | Sugar-containing beverages | | Times/d | | >2/day vs. <2/day | | Initial/manifest dental caries  (Koch, 1967) at 2.5 & 3.5yr | | Present or absent | OR = 1.79 (CI = 1.00, 3.15), P = 0.045 | | | Serious |
| Hooley 2012 ^14^ | 4.79 y | | 2.05 y | | 4149 | | 24-h dietary recall | Sweet drinks | | Frequency/d | | Continuous | | Dental caries (reported by primary caregiver) at 6-7 y and 8-9 y | | Yes or No to cavities, extractions or fillings since last survey | 2 y: OR = 1.02 (SE = 0.03), P = 0.56; 4 y OR = 1.10 (SE = 0.04) P = 0.01 | | | Serious |
| Ismail 2008 ^15^ | 30 mo | | 12 mo | | 692 | | Questionnaire completed by parents at 1, 2.5 and 3.5 y | Soda beverages | | Frequency/d | | ≥2 vs.<2/d | | Initial/manifest dental caries | | Present or absent | OR = 1.79 (CI = 1.00, 3.15), P = 0.045 | | | Serious |
|  | 0-5 y | | 2 y | | 788 | | FFQ | Soda beverages | | Frequency/wk | | Continuous | | ECC; Severe ECC | | Present or absent | Caries: OR = 1.27 (SD = 0.20) P = 0.14; Severe caries OR = 0.13 (SD = 0.13), P = 0.04 | | | Serious |
| Mei 2021^16^ | 3-4 y | | 2 y | | 549 | | Parental questionnaire on dietary intake | Carbonated beverages; Sugary drinks/snacks at night | | Frequency/d | | 1 vs <1 time/day and >1 vs <1 time/day; Never vs sometimes; never vs always | | dmft and dmfs (WHO, 1997) | | dmft; ECC | SSB: dmft (univariable) 1/day: b=0.21 [−0.71, 1.14]; p=0.651; >1 /day: b=0.45 (−0.89, 1.79) vs <1 /day P=0.510; ECC (univariable) 1/day: OR=1.19 [0.70, 2.02]; P=0.523; >1 /day: OR=1.77 (0.81, 3.89) vs <1 /day p=0.155; Sugary drinks/snacks at night: dmft: Sometimes vs never: b = 0.88 (95% CI = 0.20, 1.56), P = 0.011; always vs never: b = 1.19 (95% CI = 0.13, 2.25). P = 0.028; ECC: sometime vs never: OR = 1.29, (95% CI = 0.90, 1.84), P = 0.163, always vs never: OR = 1.42, (95% CI = 0.86, 2.36), P = 0.168 | | | Serious |
| Pang 2015 ^17^ | 3-6 y | | 2 y | | 887 | | Questionnaire completed by parents | SSB | | Frequency/d | | ≥ 1/d vs.< 1/d; ≥ 1/d vs.< 1/d | | DMFT/dmft caries | | New cases of caries | OR = 3.73 (95% = 1.55, 8.97) | | | Serious |
| Skafida 2018 ^18^ | 2 y | | 3 y | | 3770 | | FFQ | SSB | | Frequency/mo | | Several times/mo vs.< once/mo or never | | Decayed, extracted or filled teeth | | Dichotomous (0, 1) | OR = 1.26 (95% CI = 1.01, 1.55), P < 0.05 | | | Serious |
| Tamaki 2009 ^19^ | 5 or 6 y | | 2.5 y | | 500 | | FFQ | SSB | | Frequency/d | | Continuous | | Incident caries | | Change from baseline to follow-up | OR = 1.355, (95% CI = 0.963, 1.908), P = 0.08 | | | Critical |
| Thornley 2021 ^20^ | 2 y | | 5 y | | 4111 | | FFQ | SSB | | Frequency/mo | | Four groups | | dmft | | Category based on dmft score | Univariate analysis: P < 0.001 | | | Critical |
| 5-<10 y |  | |  | |  | |  |  | |  | |  | |  | |  |  | | |  |
| Lin 2021 ^21^ | 8-9 y | | 1 y | | 494 | | Parental questionnaire | Carbonated drinks; Handmade drinks | | Frequency/week; sweetness | | Often vs seldom; Sugar rich vs non-sugary | | Incidence caries | | Incidence from baseline to 1 y follow up | Carbonated drinks: OR = 1.9 (95% CI = 1.0, 3.7) P < 0.01;  Handmade: OR = 1.7 (95% CI = 1.1, 2.9), P < 0.05; OR = 0.8 (95% CI = 0.7, 1.0), P > 0.05 | | | Serious |
| **Exposure: Unhealthy foods** | |  | |  | |  | | |  | |  | |  |  |  | | |  |  |  |
| 0–<2 y |  | |  | |  | |  |  | |  | |  | |  | |  |  | | |  |
| Anderson 2021 ^2^ | 1 y | | 6 y | |  | | Questionnaire completed by parents with examiner | Sweets | | Frequency/week | | Not consumed vs consumed ≥ 1/day at 1,2 and 3 y old | | ICDAS | | Dental caries (defs 0 vs >0)_at 1, 2, 3, 5 and 7 y. | OR=1.65 (95% CI = 1.14, 2.38) (2 y); OR=2.06 (95% CI = 1.63, 2.60 (3 y); OR = 1.98 (95% CI = 1.54, 2.55) (5 yr); OR=1.88 (95% CI = 1.46, 2.41) (7 y) | | | Serious |
| Chaffee 2015 ^22^ | 6 mo | | 32 mo | | 458 | | Parent interview on age of introduction of child foods | 6 mo sweet index; 12 mo sweet index | | Time of introduction | | 6 mo sweet index Tertile 3 vs.1; 12 mo sweet index Tertile 3 vs. Tertile 1 | | Severe ECC; dmft | | ≥1 affected maxillary anterior teeth or ≥4 decayed, missing due to caries, or restored tooth surfaces | Upper vs. lowest tertile for: severe ECC at 6 mo: RR = 1.46 (95% CI = 0.97, 2.04) at 12 mo: RR = 1.55 (95% CI = 1.17, 2.23) (cumulative incidence ratio); dmft at 6 mo: RR = 1.62 (95% CI = 0.91, 2.70), dmft at 12 mo RR = 1.78 (95% CI = 1.20, 2.90) | | | Serious |
| Devenish 2020 ^23^ | 3 mo | | 26 mo | | 965 | | 24-h recall, 2-d food diary at 1 y; FFQ at 2 y | Energy as free sugars | | %EI | | > 10% EI free sugar vs. <5%EI free sugar at 1 and 2 y; > 10% EI as free sugar at 1 or  2 y (not both) vs.<5% EI free sugar at 1 and 2 y; <10% EI free sugar on at 1 and 2 y, but >5 % at least at 1 or 2 y vs.<5% EI free sugar at 1 and 2 y | | Presence of ECC | | Present or absent | Prevalence ratio = 1.97 (95% CI = 1.13, 3.44) | | | Serious |
| Feldens 2010 ^24^; 2021^25^ | 6 mo | | 44.5 mo | | 340 | | Face-to-face structured interviews | High density of sugar | | Consumed or not consumed | | Consumed vs. not consumed | | Severe ECC at 4 y | | dmfs | RR = 1.43 (95% CI = 1.08, 1.89), P = 0.005 | | | Moderate |
|  | 6 mo (sugary food purchase), 3 y (household sugar purchase) | | 5 y 6 mo | | 233 | | Maternal interview | Sugar-containing items; monthly sugar purchased per person | | Index; kg | | ≤3, 4-6, ≥7;  ≤ median vs  > median | | DMFT | | Presence vs absence | OR = 2.37 (95% CI = 1.02, 1.59), P = 0.036; OR = 1.62 (95% CI = 0.78, 3.36), P = 0.198 | | | Serious |
| Lopes-Gomes 2021 ^26^ | 1-3 y | | 3 y 9 mo | | 137 | | Maternal questionnaire on daily snacks containing sugar | Sugar-containing snacks | | Frequency/d | | ≤2/day at baseline to >2/day; >2/day at baseline to >2/day | | ICDAS | | Dental caries incidence | RR = 1.67 (95% CI 1.09, 2.52); RR = 1.81 (95% CI = 1.14, 2.87) | | | Serious |
| MacKeown 2000 ^27^ | 1 y | | 4 y | | 259 | | Semi-quantitative FFQ | Added sugar | | g/d (continuous) | | Continuous | | dmfs incidence | | dmfs score change from age 1 to 5 y | Not significant | | | Critical |
| Manohar, 2021 ^28^ | 4 mo | | 39 mo | | 718 | | 7 day food diary at 4 mo, 8 mo, 1 y, 2 yr, 3 y | Sugary foods | | Diet trajectory | | Low and gradual rising vs moderate and stable ; low and gradual rising vs high and late declining ; overall trend | | dmfs | | Dental caries at 3-4 y | IRR = 1.30, (95% CI = 0.85, 2.0), P = 0.228 ; Highest vs lowest IRR = 0.90, (95% CI = 0.47, 1.70), P = 0.019 ; overall P = 0.737 | | | Moderate |
| Mattila 2001 ^29^ | 18 mo | | 8.5 y | | 413 | | Semi structured questionnaire | Sweets/candy | | Frequency/wk | | Daily or a couple of times a week vs. more seldom; Once/week vs. more seldom | | dmft/DMFT score at 10 y | | Score at 10 y | Daily/two times/wk OR = 5.5 (95% CI = 1.9, 15.8) ; once/wk OR = 2.4 (95% CI = 0.8, 7.6) | | | Serious |
| Meurman 2010 ^30^ | 18 mo | | 24 mo | | 366 | | Dietary recall questionnaire | Added sugar; sweets snacks | | Frequency/wk | | Added sugar vs. never; Sweet snacks vs. never/seldom | | dmfs | | Caries increment (dichotomous) 18 m to 5 y | Added sugar OR = 2.2, (95% CI = 1.1, 4.5), P = 0.024; Sweet snacks OR = 1.7 (95% CI = 0.8, 3.9), P = 0.169 | | | Serious |
| Peres 2016 ^31^ | 1 mo | | 18 y | | 302 | | FFQ | Sugar intake | | Frequency/d | | High sugar intake vs. low intake (≥2 of ages 4, 15, and 18 y have been measured low intake); Upward sugar intake vs. low intake (≥2 of ages 4, 15, and 18 y have been measured low intake) | | dmft score | | Prevalence and mean dmft score | High: IRR = 1.67 (95% CI = 1.23, 2.25), upward: IRR = 1.22 (95% CI = 0.94, 1.59) | | | Moderate |
| Sakuma 2007 ^32^ | 1.5 y | | 1.5 y | | 5107 | | FFQ | Sweets/candy | | Frequency/d | | Continuous | | Change in caries | | Change in number of teeth with caries | Four cities/districts: β = 0.31, OR = 1.4 (CI = 1.2, 1.5) P < 0.001; β = 0.33, OR = 1.4 (CI = 1.1, 1.7), P < 0.1; β = 0.5, OR = 1.6 (CI = 1.3, 2.1), P < 0.001; β = 0.37, OR = 1.5 (CI = 1.3, 1.7), P < 0.001 | | | Serious |
| Watanabe 2014 ^11^ | 1.5 y | | ~21 mo | | 31202 | | FFQ | Sweet snacks | | Frequency/d | | Daily consumption vs. not consumed: 1 d/wk vs.0 d/wk; 2 d/wk vs.0 d/wk; 3 d/wk vs.0 d/wk | | Dental caries present or absent | | Dichotomous (0, 1) | Once/d (AOR: 2.0 (95% CI: 1.46, 2.74), P < 0.001); twice/d AOR = 3.21 (95% CI = 2.34, 4.40); 3 times/d AOR = 3.90 (95% CI = 2.79, 5.45) vs. none at 1.5 y | | | Serious |
| 2–<5 y |  | |  | |  | |  |  | |  | |  | |  | |  |  | | |  |
| De Melo 2019 ^33^ | 30 mo | | - | | 469 | | Questionnaire | Sweets/candy | | Never, sometimes, daily | | Sweets daily vs. never; Sweets sometimes vs. never | | dmft index | | Increase index from 18-36 mo | Daily: RR = 1.53 (95% CI = 1.09,2.14), P = 0.014; sometimes: RR = 1.12 (95% CI = 0.79, 1.58), P = 0.527 | | | Serious |
| Grindefjord 1996 ^13^ | 30 mo | | 12 mo | | 692 | | Questionnaire completed by parents at 1, 2.5 and 3.5 y | Candy | | Times/d | | ≥1 vs. <1/wk | | Initial/manifest dental caries  (Koch, 1967) at 2.5 & 3.5 y | | Present or absent | OR = 1.63 (CI = 1.04, 2.55), P = 0.032 | | | Serious |
| Hao 2015 ^34^ | 3 y | | 12 mo | | 130 | | FFQ | Sweets/candy | | Frequency/d | | Sweets ≥2 times/d vs.< 2 times/d | | dmfs at 6 mo; dmfs at 12 mo | | Present or absent | P < 0.01; P < 0.01 | | | Serious |
| Holt 1991^35^ | 2 y | | 3 y | | 2139 | | Questionnaire | Sweetened snacks or drinks | | Frequency/d | | 0, 1, 2, 3 or 4 | | dmft count | | Mean dmft | Univariate analysis: % caries free by intake (unadjusted P < 0.05), number of caries by intake (P < 0.01) | | | Critical |
| Hooley 2012 ^14^ | 4.79 y | | 2.05 y | | 4149 | | 24-h dietary recall | High fat foods (meat pie, hamburger, hot dog, sausage, or sausage roll; hot chips or French fries; potato chips or savory snacks and biscuits, doughnuts, cake, pie, or chocolate) | | Frequency/d | | Continuous | | Dental caries (reported by primary caregiver) at 6-7 y and 8-9 y | | Yes/No to occurrence of cavities, extractions or fillings since last survey | At 6-7 y OR = 1.10 (SE = 0.04), P = 0.02; at 8-9 y OR = 1.13 (SE = 0.06), P = 0.01 at 4 y | | | Serious |
| Mei 2021^16^ | 3-4 y | | 2 y | | 549 | | Parental questionnaire on dietary intake | Sweet snacks | | Frequency/d | | 1 time/day vs <1 time/day and >1 time/day vs <1 time/day | | dmft and dmfs (WHO, 1997) | | dmft; ECC | dmft: 1/day vs <1/day b = 0.28 (95% CI = -0.33, 0.89), P = 0.374; >1/day vs <1/day b = 0.89 (95% CI -0.15, 1.94) P = 0.095. ECC 1/day vs <1/day OR = 1.21 (95% CI 0.90, 1.63) P = 0.215; >1/day vs <1/day OR = 1.86 (95% CI 1.06, 3.27) P = 0.03 | | | Serious |
| Pang 2015 ^17^ | 3-6 y | | 2 y | | 887 | | Questionnaire completed by parents | Cookies and sweet breads | | Frequency/d | | ≥ 1/d vs.< 1/d; ≥ 1/d vs.< 1/d | | DMFT/dmft caries | | New cases of caries | OR = 2.01 (95% CI = 1.39, 2.92) | | | Serious |
| Peltzer 2014 ^36^ | 24 mo | | 12 mo | | 597 | | Diary completed by parents | Sweet candy | | Frequency/wk | | Weekly sweet candy intake at 30 mo: 3-7 d/wk vs.0-2 d/wk | | dmft and dmfs | | dmft value at age 36 mo minus that at 24 mo | OR = 1.97 (95% CI = 1.17, 3.31), P < 0.05 | | | Critical |
| Rodrigues 2000 ^37^ | 3 y | | 12 mo | | 510 | | 3-d weighed inventory Frequency of consumption | Sugary food | | Frequency/d | | 4-5 times/d vs.1-2.9 times/d | | Change in dmfs | | Score | OR = 4.29 (95% CI = 1.7, 10.7) | | | Moderate |
| Ruottinen 2004 ^38^ | 37.4 mo | | 6 y | | 89 | | 4-d food record | Added sucrose (sucrose and other free sugars); daily sugar | | % EI | | <10% EI vs. ≥10 % EI; continuous | | dmft/DMFT | | Change in score | Mean = 2.82 (SEM = 0.51) v 1.63 (SEM = 0.26) P = 0.014; P = 0.012 | | | Serious |
| Skafida 2018 ^18^ | 2 y | | 3 y | | 3770 | | FFQ | Sweets or chocolate | | Frequency/mo | | ≥1/d vs. < 1/d | | Decayed, extracted, or filled teeth | | Dichotomous (0, 1) | OR = 1.53 (95% CI = 1.24, 1.89) P < 0.001 | | | Serious |
| Tamaki 2009 ^19^ | 5 or 6 y | | 2.5 y | | 500 | | FFQ | Sweet snacks | | Frequency/d | | Continuous | | Incident caries (baseline to follow-up) | |  | OR = 1.286 (95% CI = 0.822, 2.013) P = 0.271 | | | Critical |
| Thornley 2021 ^20^ | 2 y | | 5 y | | 4111 | | FFQ | Confectionary/cakes; noodles/rice porridge; ice-cream; takeaways | | Frequency/mo | | Continuous | | dmft | | Score | Univariate analysis: Confectionary or cake P < 0.001; Noodles or rice porridge P < 0.001; Ice-cream P < 0.001; Refined breakfast cereals P < 0.001; Takeaways P < 0.001 | | | Critical |
| Winter 2015 ^39^ | 3.5 y | | 3 y | | 566 | | Questionnaire | Sugar index | | Frequency: never, seldom, occasionally,  often, always | | Above vs. below median score (>24 vs. ≤ 24) | | dmft increment | | Incremental change | OR = 1.53 (95% CI = 1.07, 2.2) P = 0.027 | | | Critical |
| Wu 2020 ^40^ | 4.2 y | | 1 y | | 212 | | Questionnaire | Candy | | Frequency/wk | | > 1/wk vs. <1/wk | | dmft rate | | Score | Parameter estimate = -3.093 (95% CI = -1.095, -0.242), P = 0.004 | | | Serious |
| >5 – 10 y |  | |  | |  | |  |  | |  | |  | |  | |  |  | | |  |
| Mahboobi 2021 ^41^ | 7-8 y | | 2 y | | 290 | | 3-day food record | Sugary snacks | | Frequency/day | | ≥2 vs <2 | | CAST index at 2 y | | Dental caries | 38% vs 62% IRR = 0.96 (95% CI = 0.73, 1.27), P = 0.80 | | | Moderate |

*Estimates are adjusted odds ratios unless otherwise stated

^†^AOR, adjusted OR; CAST index, Caries Assessment Spectrum and Treatment; dmfs, decayed-missing-filled surfaces (for primary teeth); DAT, dietary assessment tool; dmft, decayed-missing-filled teeth (for primary teeth); DMFT, decayed-missing-filled teeth (for permanent teeth); d1, non-cavitated lesions; d2-3, cavitated lesions; ECC, early childhood caries; FFQ, food-frequency questionnaire; RR = Relative risk; RoB, risk of bias; SSB, sugar-sweetened beverages; %EI, percentage of energy intake; IRR, Incidence rate ratio.

^‡^Minimum analytical sample size.

**Figure S1** Individual risk of bias assessment for non-randomized studies reporting the effect of unhealthy food and beverage consumption on dental caries in children aged ≤10 years using ROBINS-I


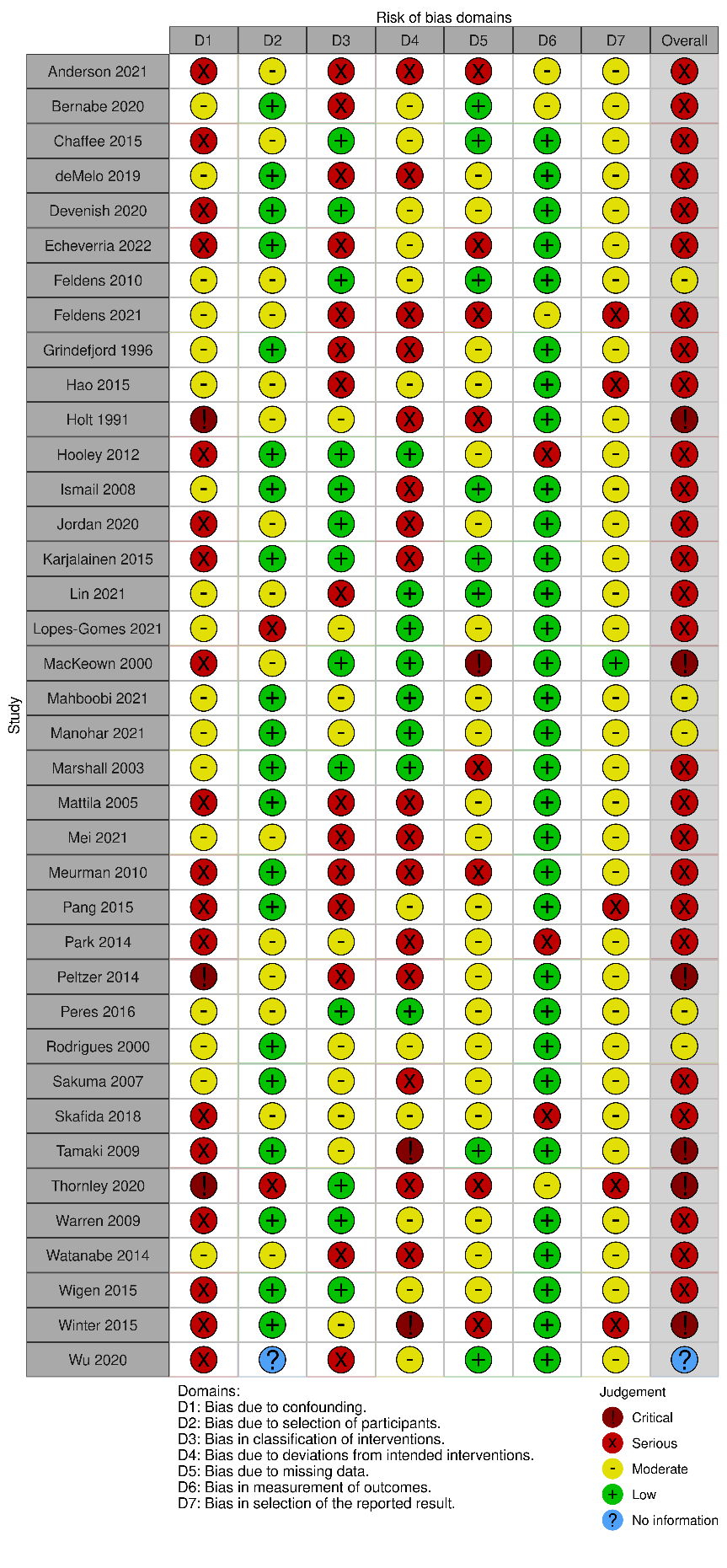


**Figure S2** Summary risk of bias across included studies on the effect of unhealthy food and beverage consumption on dental caries in children aged ≤10 years


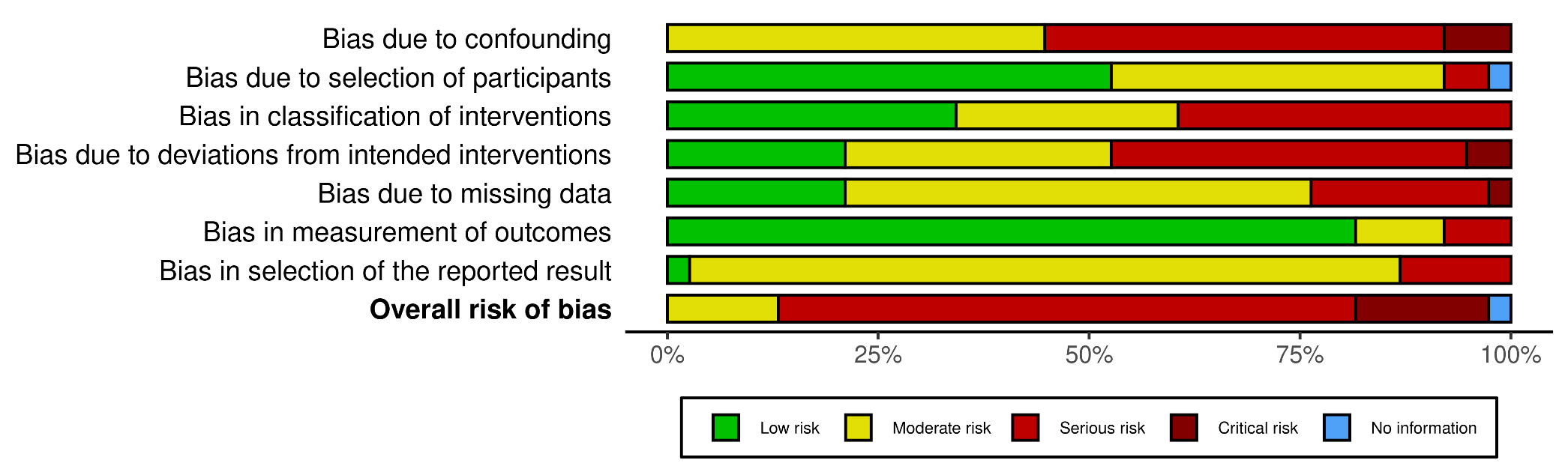


**References**

1. Sterne J, Hernán M, Reeves B, et al. Risk Of Bias In Non-randomized Studies of Interventions (ROBINS-I): detailed guidance. Bmj.

2. Anderson M, Dahllöf · G, Warnqvist · A, Grindefjord · M. Development of dental caries and risk factors between 1 and 7 years of age in areas of high risk for dental caries in Stockholm, Sweden. *European Archives of Paediatric Dentistry* . 2021;22(5):947-957. doi:10.1007/s40368-021-00642-1

3. Bernabé E, Ballantyne H, Longbottom C, Pitts NB. Early Introduction of Sugar-Sweetened Beverages and Caries Trajectories from Age 12 to 48 Months. *J Dent Res*. 2020;99(8):898-906. doi:10.1177/0022034520917398

4. Echeverria MS, Schuch HS, Cenci MS, et al. Trajectories of Sugar Consumption and Dental Caries in Early Childhood. *J Dent Res*. 2022;101(6):724-730. doi:10.1177/00220345211068743

5. Jordan KH, McGwin G, Childers NK. Children’s detailed non-water beverage consumption habits and longitudinal early childhood caries experiences. *J Public Health Dent*. 2020;80(4):271-277. doi:10.1111/jphd.12381

6. Marshall TA, Levy SM, Broffitt B, et al. Dental caries and beverage consumption in young children. *Pediatrics*. 2003;112(3 Pt 1). doi:10.1542/peds.112.3.e184

7. Levy SM, Warren JJ, Broffitt B, Hillis SL, Kanellis MJ. Fluoride, beverages and dental caries in the primary dentition. *Caries Res*. 2003;37(3):157-165. doi:10.1159/000070438

8. Park S, Lin M, Onufrak S, Li R. Association of Sugar-Sweetened Beverage Intake during Infancy with Dental Caries in 6-year-olds. *Clin Nutr Res*. 2015;4(1):9. doi:10.7762/cnr.2015.4.1.9

9. Sakuma S, Nakamura M, Miyazaki H. Predictors of dental caries development in 1.5-year-old high-risk children in the Japanese public health service. *J Public Health Dent*. 2007;67(1):14-19. doi:10.1111/j.1752-7325.2007.00003.x

10. Warren JJ, Weber-Gasparoni K, Marshall TA, et al. A longitudinal study of dental caries risk among very young low SES children. *Community Dent Oral Epidemiol*. 2009;37(2):116-122. doi:10.1111/j.1600-0528.2008.00447.x

11. Watanabe M, Wang DH, Ijichi A, et al. The Influence of Lifestyle on the Incidence of Dental Caries among 3-Year-Old Japanese Children. *Int J Environ Res Public Health*. 2014;11(12):12611-12622. doi:10.3390/ijerph111212611

12. Wigen TI, Wang NJ. Does early establishment of favorable oral health behavior influence caries experience at age 5 years? *Acta Odontol Scand*. 2015;73(3):182-187. doi:10.3109/00016357.2014.976264

13. Grindefjord M, Dahllof G, Nilsson B, Modeer T. Stepwise prediction of dental caries in children up to 3.5 years of age. *Caries Res*. 1996;30:256-266.

14. Hooley M, Skouteris H, Millar L. The relationship between childhood weight, dental caries and eating practices in children aged 4-8 years in Australia, 2004-2008. *Pediatr Obes*. 2012;7(6):461-470. doi:10.1111/j.2047-6310.2012.00072.x

15. Ismail AI, Lim S, Dohn W, Willem J. Determinants of early childhood caries in low-income African American young children. *Pediatr Dent*. 2008;30(4):289-296. doi:10.1016/0090-4295(93)90520-k

16. Mei L, Shi H, Wei Z, Li Q, Wang X. Risk factors associated with early childhood caries among Wenzhou preschool children in China: a prospective, observational cohort study. *BMJ Open*. 2021;11(9):e046816. doi:10.1136/bmjopen-2020-046816

17. Pang M, Zeng X, Tang Q. A study of dental caries and risk factors in children of Guangxi area. *Shanghai Journal of Stomatology*. 2015;24(5):611-615.

18. Skafida V, Chambers S. Positive association between sugar consumption and dental decay prevalence independent of oral hygiene in pre-school children: a longitudinal prospective study. *J Public Health (Oxf)*. 2018;40(3):e275-e283. doi:10.1093/pubmed/fdx184

19. Tamaki Y, Nomura Y, Katsumura S, et al. Construction of a dental caries prediction model by data mining. *J Oral Sci*. 2009;51(1):61-68. doi:10.2334/josnusd.51.61

20. Thornley S, Bach K, Bird A, et al. What factors are associated with early childhood dental caries? A longitudinal study of the *Growing Up in New Zealand* cohort. *Int J Paediatr Dent*. 2021;31(3):351-360. doi:10.1111/ipd.12686

21. Lin PY, Lee YC, Hsu LY, Chang HJ, Chi LY. Association between sugary drinks consumption and dental caries incidence among Taiwanese schoolchildren with mixed dentition. *Community Dent Oral Epidemiol*. 2022;50(5):384-390. doi:10.1111/cdoe.12683

22. Chaffee BW, Feldens CA, Rodrigues PH, Vítolo MR. Feeding practices in infancy associated with caries incidence in early childhood. *Community Dent Oral Epidemiol*. 2015;43(4):338-348. doi:10.1111/cdoe.12158

23. Devenish G, Mukhtar A, Begley A, et al. Early childhood feeding practices and dental caries among Australian preschoolers. *American Journal of Clinical Nutrition*. 2020;111(4):821-828. doi:10.1093/ajcn/nqaa012

24. Feldens CA, Giugliani ERJ, Vigo Á, Vítolo MR. Early feeding practices and severe early childhood caries in four-year-old children from southern Brazil: A birth cohort study. *Caries Res*. 2010;44(5):445-452. doi:10.1159/000319898

25. Feldens CA, Dos Santos IF, Kramer PF, Vítolo MR, Braga VS, Chaffee BW. Early-Life Patterns of Sugar Consumption and Dental Caries in the Permanent Teeth: A Birth Cohort Study. *Caries Res*. 2021;55(5):505-514. doi:10.1159/000518890

26. Lopes-Gomes R, Letícia Ramos-Jorge M, Fernandes IB, Vieira EM, Pordeus IA, Ramos-Jorge J. Untreated dental caries and visible plaque of mothers are not determinant for the incidence of caries in dentin among children: evidence from a 3-year prospective cohort study. *Clinical Oral Investigations* . 2021;25:5431-5439. doi:10.1007/s00784-021-03851-6/Published

27. MacKeown JM, Cleaton-Jones PE, Edwards AW. Energy and macronutrient intake in relation to dental caries incidence in urban black South African preschool children in 1991 and 1995: The birth-to-ten study. *Public Health Nutr*. 2000;3(3):313-319. doi:10.1017/s1368980000000355

28. Manohar N, Hayen A, Scott JA, et al. Impact of Dietary Trajectories on Obesity and Dental Caries in Preschool Children: Findings from the Healthy Smiles Healthy Kids Study. *Nutrients*. 2021;13(7):2240. doi:10.3390/nu13072240

29. Mattila ML, Rautava P, Paunio P, et al. Caries Experience and Caries Increments at 10 Years of Age. *Caries Res*. 2001;35(6):435-441. doi:10.1159/000047487

30. Meurman PK, Pienihäkkinen K. Factors associated with caries increment: A longitudinal study from 18 months to 5 years of age. *Caries Res*. 2011;44(6):519-524. doi:10.1159/000320717

31. Peres MA, Sheiham A, Liu P, et al. Sugar consumption and changes in dental caries from childhood to adolescence. *J Dent Res*. 2016;95(4):388-394. doi:10.1177/0022034515625907

32. Sakuma S, Nakamura M, Miyazaki H. Predictors of dental caries development in 1.5-year-old high-risk children in the Japanese public health service. *J Public Health Dent*. 2007;67(1):14-19. doi:10.1111/j.1752-7325.2007.00003.x

33. De Melo MMDC, De Souza WV, De Goes PSA. Increase in dental caries and change in the socioeconomic profile of families in a child cohort of the primary health care in Northeast Brazil. *BMC Oral Health*. 2019;19(1):1-10. doi:10.1186/s12903-019-0871-9

34. Hao W, Xu H, Chen X, et al. Changes in dental plaque microbial richness and oral behavioral habits during caries development in young chinese children. *Caries Res*. 2015;49(2):116-123. doi:10.1159/000366505

35. Holt R. Foods and drinks at four daily time intervals in a group of young children. *Br Dent J*. 1991;170(4):137-143.

36. Peltzer K, Mongkolchati A, Satchaiyan G, Rajchagool S, Pimpak T. Sociobehavioral factors associated with caries increment: a longitudinal study from 24 to 36 months old children in Thailand. *Int J Environ Res Public Health*. 2014;11(10):10838-10850. doi:10.3390/ijerph111010838

37. Rodrigues CS, Sheiham A. The relationships between dietary guidelines, sugar intake and caries in primary teeth in low income Brazilian 3-year-olds: A longitudinal study. *Int J Paediatr Dent*. 2000;10(1):47-55. doi:10.1046/j.1365-263x.2000.00165.x

38. Ruottinen S, Karjalainen S, Pienihäkkinen K, et al. Sucrose intake since infancy and dental health in 10-year-old children. *Caries Res*. 2004;38(2 PG-142-8):142-148. doi:10.1159/000075938

39. Winter J, Glaser M, Heinzel-Gutenbrunner M, Pieper K. Association of caries increment in preschool children with nutritional and preventive variables. *Clin Oral Investig*. 2015;19(8):1913-1919. doi:10.1007/s00784-015-1419-2

40. Wu R, Cao G, Feng V, Feng X, Chen X, Han X. Risk factors of dental caries among young children in Pudong New District, Shanghai. *Shanghai Journal of Stomatology*. 2020;29(4).

41. Mahboobi Z, Pakdaman A, Yazdani R, Azadbakht L, Shamshiri AR, Babaei A. Caries incidence of the first permanent molars according to the Caries Assessment Spectrum and Treatment (CAST) index and its determinants in children: a cohort study. *BMC Oral Health* . 2021;21(1):1-10. doi:10.1186/s12903-021-01612-1
