# Supplementary material for: Impact of unhealthy food and beverage consumption on children’s risk of dental caries: a systematic review
Source: Nutr Rev. 2023 Dec 12;82(11):1539–55. doi: 10.1093/nutrit/nuad147 (PMC11465133; doi:10.1093/nutrit/nuad147)
Supplement: nuad147_Supplementary_Data [file nuad147_supplementary_data.zip › nuad147_Supplementary_Data/Title Page.docx]

**Article type: Systematic review**

**Impact of unhealthy food and beverage consumption in children on risk of dental caries: a systematic review**

Authors: Jessica F Large^1^ 0000-0002-5092-7088, Claire Madigan^1^ 0000-0002-6782-0017, Rebecca Pradeilles^2,3^ 0000-0003-0334-3714, Oonagh Markey^2^ 0000-0001-5293-7354, Benjamin Boxer^2^, Emily K Rousham^2^ 0000-0001-5654-9279

^1^Centre for Lifestyle Medicine and Behaviour, School of Sport, Exercise and Health Sciences

National Centre for Sport and Exercise Medicine, Loughborough University, UK

^2^ School of Sport, Exercise and Health Sciences, Loughborough University, UK.

^3^ UMR MoISA (Montpellier Interdisciplinary Centre on Sustainable Agri-Food Systems), (Univ Montpellier, CIRAD, CIHEAM-IAMM, INRAE, Institut Agro, IRD), Montpellier, France

Key words: unhealthy foods, sugar sweetened beverages (SSBs), dental caries, children, systematic review, ultra-processed foods

Running title: Unhealthy foods and dental caries

Acknowledgements

We gratefully thank the following for assistance in searches, screening or document retrieval for the wider systematic review: Kathrin Burdenski (KB), Chris Carroll, Sophie Goudet (SG), Paula Griffiths (PG), Natalie Pearson (NP), Nathan Rush, Megan Stanley (MS) and Yvanna Todorova.

JFL and CM work at the Centre for Lifestyle Medicine and Behaviour, Loughborough University under Professor Amanda Daley (AJD) who is supported by a National Institute for Health Research (NIHR) Research Professorship award. The views expressed are those of the authors and not necessarily those of the NHS, the NIHR or the Department of Health and Social Care.

Author Contributions

EKR had primary responsibility for the conception and design of the study. All authors contributed to data collection or analysis, writing or revision, and final reading and approval of the manuscript. JL and EKR had primary responsibility for writing the manuscript.

Funding

Funding support was received from the Food and Nutrition Action in Health Systems unit, Department of Nutrition and Food Safety, World Health Organization (EKR, RP). The funders had no role in the collection, analysis, interpretation of data or writing of the manuscript.

Declaration of interests

The authors declare no conflicts of interest.

Correspondence

Jessica F Large, The Centre for Sport and Exercise Medicine, School of Sport, Exercise and Health Sciences, NCSEM 2.22, Epinal Way, Loughborough University, Loughborough, LE11 3TU. Telephone: 01509 226372. Email: [j.large@lboro.ac.uk](mailto:j.large@lboro.ac.uk)

Systematic review registration number: CRD42020218109
